# In‐cytoplasm mitochondrial transplantation for mesenchymal stem cells engineering and tissue regeneration

**DOI:** 10.1002/btm2.10250

**Published:** 2021-09-28

**Authors:** Xudong Yao, Yuanzhu Ma, Wenyan Zhou, Youguo Liao, Zongsheng Jiang, Junxin Lin, Qiulin He, Hongwei Wu, Wei Wei, Xiaozhao Wang, Mikael Björklund, Hongwei Ouyang

**Affiliations:** ^1^ Dr. Li Dak Sum & Yip Yio Chin Center for Stem Cells and Regenerative Medicine, Second Affiliated Hospital Zhejiang University School of Medicine Hangzhou China; ^2^ Zhejiang University‐University of Edinburgh Institute (ZJU‐UoE Institute), Zhejiang University Haining China; ^3^ The Fourth Affiliated Hospital Zhejiang University School of Medicine Yiwu China; ^4^ Department of Sports Medicine Zhejiang University School of Medicine Hangzhou China; ^5^ China Orthopedic Regenerative Medicine Group (CORMed) Hangzhou China; ^6^ Key Laboratory of Tissue Engineering and Regenerative Medicine of Zhejiang Province, Zhejiang University School of Medicine Hangzhou China

**Keywords:** bioenergetics, mesenchymal stem cells, mitochondrial transfer, tissue regeneration

## Abstract

Stem cell therapies are unsatisfactory due to poor cell survival and engraftment. Stem cell used for therapy must be properly “tuned” for a harsh in vivo environment. Herein, we report that transfer of exogenous mitochondria (mito) to adipose‐derived mesenchymal stem cells (ADSCs) can effectively boost their energy levels, enabling efficient cell engraftment. Importantly, the entire process of exogeneous mitochondrial endocytosis is captured by high‐content live‐cell imaging. Mitochondrial transfer leads to acutely enhanced bioenergetics, with nearly 17% of higher adenosine 5′‐triphosphate (ATP) levels in ADSCs treated with high mitochondrial dosage and further results in altered secretome profiles of ADSCs. Mitochondrial transfer also induced the expression of 334 mRNAs in ADSCs, which are mainly linked to signaling pathways associated with DNA replication and cell division. We hypothesize that increase in ATP and cyclin‐dependent kinase 1 and 2 expression might be responsible for promoting enhanced proliferation, migration, and differentiation of ADSCs in vitro. More importantly, mito‐transferred ADSCs display prolonged cell survival, engraftment and horizontal transfer of exogenous mitochondria to surrounding cells in a full‐thickness skin defect rat model with improved skin repair compared with nontreated ADSCs. These results demonstrate that intracellular mitochondrial transplantation is a promising strategy to engineer stem cells for tissue regeneration.

**List of abbreviations**
ADSCs: adipose‐derived mesenchymal stem cells
ATP: adenosine 5′‐triphosphate
ALP: alkaline phosphatase
CDK: cyclin‐dependent kinase
CCK‐8: Cell Counting Kit‐8
Dox: doxorubicin
DLS: dynamic light scattering
FBS: fetal bovine serum
FOVs: fields of view
GO: Gene Ontology
HBSS: Hank's balanced salt solution
IGFBP6: insulin‐like growth factor binding proteins 6
LC/MS‐MS: liquid chromatography tandem mass spectrometry
MDGF: myeloid‐derived growth factor
Mito: mitochondria
MSCs: mesenchymal stem cells
mtDNA/nDNA: mitochondrial DNA/nuclear DNA
OCR: oxygen consumption rate
OXPHOS: oxidative phosphorylation
P/S: penicillin streptomycin solution
ROS: reactive oxygen species
SA‐β‐Gal: senescence associated β‐galactosidase
TEM: transmission electron microscopy
TIMP: tissue inhibitor matrix metalloproteinase
TGFBI: transforming growth factor beta induced
8‐OHdG: 8‐hydoxy 2 deoxyguanosine

## 1 INTRODUCTION

Mesenchymal stem cells (MSCs) are the so‐called “first‐generation stem cell type”. The anti‐inflammatory, immunomodulatory, angiogenic, proapoptotic and trophic activities of MSCs, in combination with the ease of isolation and amplification, have led to over 950 registered MSCs clinical trials listed with ClinicalTrials.gov. However, there are several drawbacks in using MSCs: (1) the therapeutic potential of MSCs is highly variable in a complex pathophysiological environment^1^; (2) the overall efficiency of MSCs engraftment to tissue injuries is poor, especially when these cells are systemically administered.^2^ Consequently, there is a need for innovative approaches to endow “next‐generation MSCs” with enhanced features and functionalities. Consequently, taking inspiration from chimeric antigen receptor (CAR) T cells, MSCs surface can be engineered by a IgM‐derived anti‐GD2 CAR to specifically redirect MSCs delivering proapoptotic cytokine tumor necrosis factor‐related apoptosis‐inducing ligand (TRAIL) against a GD2‐expressing tumor, in an effort to prolong site‐specific retention of MSCs.^3^ By applying popular CRISPR/Cas9‐adeno‐associated virus serotype 6 (AAV6) platform, genetically engineered MSCs lines with exogenous DNA integration overexpressing vascular endothelial growth factor and platelet‐derived growth factor provided reduced immune clearance in vivo resulting in superior therapeutic efficacy in the diabetic mouse wound healing model.^4^ However, these technologies are labor intensive and restricted with clinical applications due to genetic manipulation.

Another engineering strategy to boost the potency of MSCs is to prime the MSCs by exposing them to low oxygen cultivation,^5^ small molecules,^6^, ^7^, ^8^ functional particles,^9^, ^10^ distinct types of biomaterials,^11^, ^12^ or modified culture conditions^13^, ^14^ before transplantation. Unfortunately, the positive effects are only retained for several hours to a few days after the transfer of primed MSCs to in vivo environments.^15^ Therefore, more effective, consistent and long‐lasting priming effects are necessary.

Mitochondria are the key intracellular organelles contributing to cell energetics and viability. Since the initial discovery demonstrated that mitochondria‐deficient A549 lung cancer cells could obtain functional mitochondria from donor MSCs,^16^ the “mito‐healing” theory of mitochondrial transfer from stem cells to damaged cells has been subsequently supported by numerous studies in a variety of mammalian cells, including cardiomyocytes,^17^ neurons,^18^ immune cells,^19^, ^20^ and epithelial cells.^21^, ^22^ Remarkable elevation in adenosine 5′‐triphosphate (ATP) levels, restoration of cellular bioenergetics, and reduction in oxidative stress have been observed in the rescued recipient cells after mitochondrial transfer.^23^ The success of stem cell‐derived mitochondria to multiple cell types raises the question whether “next generation stem cells” with superior self‐functionality could be engineered by transferring additional mitochondria to MSCs. Direct mitochondrial transplantation has been explored in clinical practice. Purified respiration‐competent mitochondrial particles can be delivered via intravenous injection or intravascular coronary injection.^24^, ^25^ A successful in vivo mitochondrial transplantation trial in pediatric patients with cardiac disease in 2017 implied that mitochondria exhibit the ideal cellular organelle for transplantation, as they continue to survive independently from the host cell and further mitigate the possibility of an autoimmune reaction because of “natural attributes”.^26^

In the current study, we sought to develop a strategy for energetic engineering of MSCs by transfer of exogeneous mitochondria. Our hypothesis was that mito‐transferred MSCs would demonstrate enhanced therapeutic efficacy by modifying the metabolic state thereby positively affecting cellular phenotypes and paracrine activities. To benefit the persistent priming effects, we assembled recipient MSCs derived from human adipose tissue (ADSCs) by loading allograft mitochondrial particles isolated from donor ADSCs. We used high‐content live‐cell imaging to investigate the effective dose and the optimal timing for exogenous mitochondrial internalization. To track gene expression changes, we analyzed total RNA profiles of ADSCs modulated by exogenous mitochondria. To investigate their secretome changes associated with paracrine mechanisms, comprehensive proteomic analysis was employed by liquid chromatography tandem mass spectrometry (LC/MS‐MS). Finally, we tested the therapeutic efficacy of the mito‐transferred ADSCs by transplanting them into a rat full‐thickness skin defect model and monitoring the rate of wound healing. The results of this study pave the way for energetic engineering of “next‐generation stem cells” for tissue repair.

## 2 RESULTS

### 2.1 Isolation, purification, and characterization of isolated mitochondria from donor Y40‐ADSCs

**FIGURE 1:**
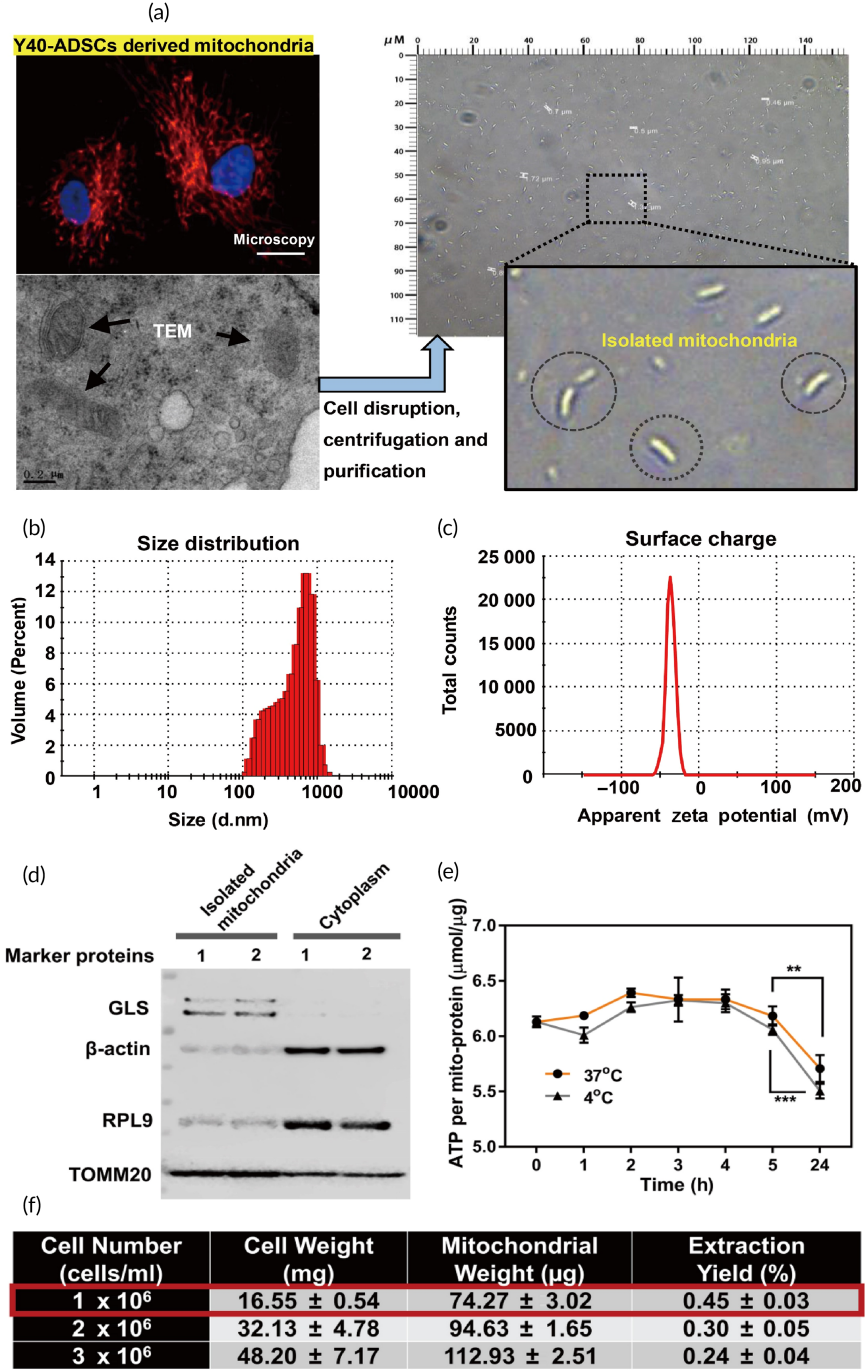
Characterization of donor Y40‐ADSCs derived mitochondria. (a) (left) Mitochondrial distribution and representative transmission electron microscopy (TEM) image of intracellular mitochondria in Y40‐ADSCs. Scale bar, 10 μm and 0.2 μm, respectively; (right) Images of isolated intact mitochondria. (b) Size distribution of isolated mitochondria. (c) Zeta potential of isolated mitochondria. (d) Western blot analysis of isolated mitochondria. TOMM20 and GLS are mitochondrial markers, RPL9 and β‐actin are cytoplasmic protein markers (three independent samples concentrated for one WB test). (e) Comparison of adenosine 5′‐triphosphate (ATP) change during the storage of isolated mitochondria at 4°C and 37°C (*n* = 8). (f) Mitochondrial isolation efficiency from different cell number (cells/ml) (*n* = 3). Significantly different (one‐way analysis of variance [ANOVA]): **p < 0.01 and ***p < 0.001

Human donor Y40‐ADSCs and recipient Y74‐ADSCs were isolated from the liposuction specimens of healthy female donors aged 40 and 74 years, respectively. The resulting ADSCs expressed specific markers for human MSCs such as CD73, CD90, and CD105 and did not express CD34 and CD45 (Figure S1a,b). Intracellular ATP levels in the Y40‐ADSCs and Y74‐ADSCs were similar (Figure S2b). To characterize mitochondria in ADSCs cells, mitochondrial DNA/nuclear DNA (mtDNA/nDNA) ratio was investigated in donor and recipient cells. There was no correlation between donor age and mtDNA/nDNA ratio (measured as ND1/SCLO2B1 and ND5/SERPINA1) (Figure S2c). Next, we marked the donor mitochondria with MitoTracker Red CMXRos while the mitochondria in recipient Y74‐ADSCs were stained with MitoTracker Green FM. Comparing mitochondria from Y40‐ADSCs and Y74‐ADSCs indicated similar tubular morphologies. Transmission electron microscopy (TEM) demonstrated the ADSCs derived mitochondria were less than 1 μm in length (Figure 1a and Figure S2a).

Excessive homogenization gives rise to mechanical damage of mitochondria. We first investigated the integrity of purified mitochondria from ADSCs. The isolated mitochondria derived from Y40‐ADSCs with differential centrifugation after mechanical lysis represented intact “rod‐shaped bacteria” appearance (Figure 1a). Further information about the mitochondrial structure formation was provided by dynamic light scattering (DLS) and zeta potential determination (Figure 1b,c). The size of the mitochondria ranged from 100 nm to approximately 1 μm, consistent with the TEM results. Moreover, the zeta potential of mitochondria was approximately −36.3 mV. The compete cell disruption was confirmed by flow cytometry (Figure S3a). The purity of mitochondria was also evaluated by western blotting to analyze the mitochondrial marker proteins in isolated mitochondria. Small cytoplasmic proteins, such as 60S ribosomal protein L9 (RPL9) and β‐actin, were present only in low levels in purified mitochondria (Figure 1d), whereas mitochondrial proteins, including glutaminase (GLS) and mitochondrial import receptor subunit TOM20 homolog (TOMM20) were enriched in mitochondrial pellet. We conclude that mitochondrial structure is retained during rapid isolation of mitochondria from ADSCs (<40 min).

To determine the biological stability, ATP changes in isolated mitochondria from Y40‐ADSCs were measured using a luciferase‐based kinetic assay over time. Mitochondrial ATP was stable within 5 h for storage at 4 or 37°C, whereas it was reduced to about 90% of initial level after 24 h (Figure 1e), indicating the difficulty of respiration competent long‐term maintenance without host cells. To meet the quantity requirement for isolated mitochondria, Y40‐ADSCs were amplified in vitro. We compared the mitochondrial yield from three different donor cell numbers. As predicted, increased cell number produced more mtDNA and mito‐protein (Figure S3b,c), while ATP production per unit of mito‐protein remained constant (Figure S3d). However, the best extraction yield (described as the percentage of isolated mitochondria by wet cell mass) was obtained using 10^6^ cells/ml (Figure 1f), possibly due to the limited capacity of Potter‐Elvehjem grinder. Taken together, isolated mitochondria from ADSCs need to be prepared in small batches and used freshly.

### 2.2 Kinetic analysis of allotransplanting exogenous mitochondria into recipient Y74‐ADSCs

**FIGURE 2:**
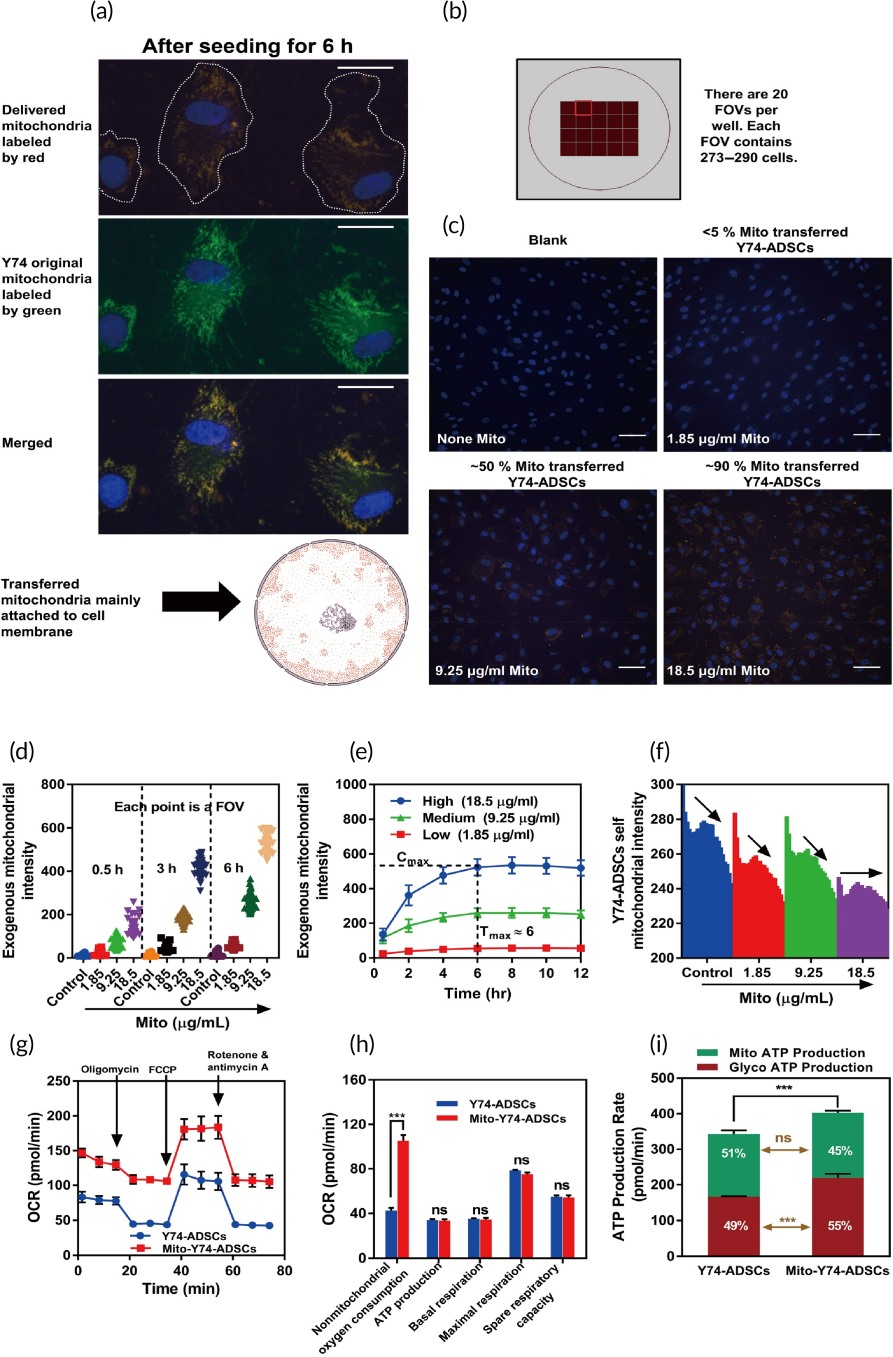
Intracellular transplantation of isolated mitochondria into recipient Y74‐ADSCs through endocytosis. (a) Intracellular localization of isolated exogenous mitochondria (red labeling) with self‐original mitochondria in Y74‐ADSCs (green labeling). Scale bar, 10 μm. (b) Mitochondrial morphology, colocalization, and mitochondrial uptake rate were visualized and quantified by live‐cell high‐content analysis. (c) The presence of exogeneous mitochondria‐positive Y74‐ADSCs after 12 h incubation. (d) Time‐dependent intracellular uptake of isolated mitochondria. (e) Dose‐dependent intracellular uptake of isolated mitochondria. (f) The tendency of exogenous mitochondria (low to high concentration with 12 h incubation) to balance mitochondrial fission/fusion dynamics in Y74‐ADSCs. (g) Oxygen consumption rates (OCRs) following the Seahorse Mito Stress Test assay in Y74‐ADSCs before and after mitochondrial transplantation. (h) The parameters within the Cell Mito Stress assay after 6 h, including mito ATP production, basal respiration, maximal respiration and spare respiratory of samples in (f). (i) Mitochondrial transplantation increased glycolytic adenosine 5′‐triphosphate (ATP) production rate in Y74‐ADSCs determined by Seahorse XF Real‐Time ATP Rate Assay after 6 h. Significantly different (one‐way analysis of variance [ANOVA]): ns, not significant and ***p < 0.001

To determine whether Y74‐ADSCs can take up exogenous Y40‐ADSCs derived mitochondria, host cells were labeled with MitoTracker Green FM. Separately, exogenous Y40‐ADSCs derived mitochondria were stained with MitoTracker Red CMXRos. If the cells take up the exogenous mitochondria, there should be intracellular localization of fluorescence signals from green‐ and red‐labeled mitochondria. Live fluorescence imaging indicated most exogenous mitochondria were found on the plasma membranes of the recipient cells after 6 h (Figure 2a).

To quantify the extent of exogenous mitochondria delivered into ADSCs, we carried out real‐time tracing of mitochondria transfer using high‐content imaging. Twenty fields of view (FOVs) were captured per well, and each FOV contained approximately 290 cells (Figure 2b). Isolated Y40‐ADSCs derived mitochondria were added at a blank, low, media, and high dose, respectively (0–18.5 μg/ml per 10^4^ recipient cells). The three‐channel fluorescence time‐lapse images were acquired over 12 h period. We found exogeneous mitochondria‐positive Y74‐ADSCs reached 90% at 18.5 μg/ml (Figure 2c), which was considered as the optimal mitochondrial dosage for further application. Dynamic mitochondrial uptake is shown as movie in [Link], [Link]. At the same time, the transport of donor mitochondria to the recipient cells showed a dose‐dependent accumulation of MitoTracker Red CMXRos‐fluorescence, which gradually increased over the first 6 h (Figure 2d). A 6‐h incubation was sufficient to reach the maximum mitochondrial concentration internalized by recipient cells (Figure 2e). Next, we used high‐throughput analysis to investigate the effects on the original mitochondria of Y74‐ADSCs. When the dose of the exogenous donor mitochondria increased, the trend in the reduction of MitoTracker Green FM—fluorescence from host mitochondria gradually slowed down during 12 h incubation (Figure 2f), suggesting that exogenous mitochondria potentially enabled to balance mitochondrial fission/fusion dynamics.

Next, we selected exogenous mitochondria at 18.5 μg/ml per 10^4^ cells with 6 h co‐culture for ADSC priming to measure the rate of respiration of mito‐transferred Y74‐ADSCs by Seahorse Bioanalyzer, which determine real‐time cellular oxygen consumption rate (OCR) changes under basal conditions responding to the addition of oligomycin (ATP synthase inhibitor), Carbonyl cyanide p‐(trifluoromethoxy) phenylhydrazone (FCCP) (mitochondrial membrane uncoupler), and rotenone (complex I inhibitor). The baseline levels of nonmitochondrial OCR were measured by rotenone and antimycin‐A (complex III inhibitor) to suppress all mitochondrial respiration (Figure 2g). The resulting parameters within the Cell Mito Stress assay, including basal respiration, mito ATP production, spare respiratory capacity, and maximal respiration represented no significant difference between control ADSCs and mito‐transferred ADSCs, as calculated by subtraction of nonmitochondrial oxygen consumption (Figure 2h). Interestingly, nonmitochondrial oxygen consumption was increased in mito‐transferred Y74‐ADSCs. Such phenomenon may suggest the existence of reactive oxygen species (ROS) (known to link with mitochondrial dysfunction). Consequently, the potential oxidative DNA damage was measured by quantifying 8‐OHdG, showing no significant change in 8‐OHdG level in mito‐transferred ADSCs compared with control (Figure S4). All this suggested the internalized exogenous mitochondria could still potential provide ATP to meet the metabolic demands, without impairing the mitochondrial function of the recipient ADSCs.

Based on the finding above, we further performed the Seahorse XF real‐time ATP rate assay to quantify metabolic switching in response to exogenous mitochondrial stimulation. A higher proportion of ATP was generated by glycolysis in mito‐transferred ADSCs than in control ADSCs (55% vs. 49%, respectively) (Figure 2i), while a smaller proportion was generated by oxidative phosphorylation (OXPHOS) (45% vs. 51%, respectively). The total ATP production rate was 17% higher in the population of mito‐transferred ADSC, suggesting that exogenous mitochondrial incorporation may increase overall metabolic activity of stem cells by glycolytic pathway, similar to the Warburg effect observed in cancer cells.

### 2.3 Improved stress tolerance of mito‐transferred Y74‐ADSCs against serum starvation and oxidative stress in vitro

**FIGURE 3:**
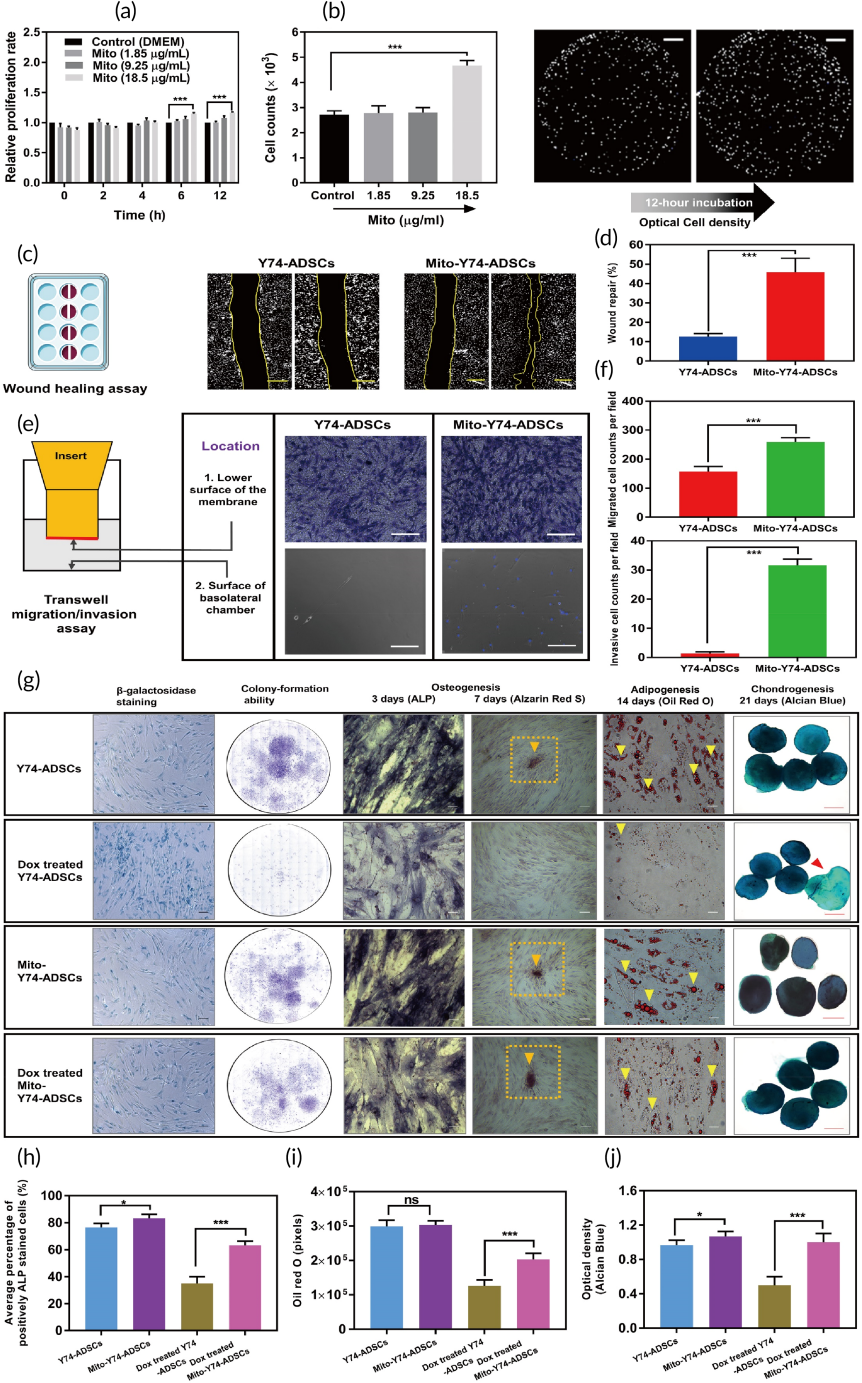
Improved stress tolerance of mito‐transferred Y74‐ADSCs against serum starvation and oxidative stress in vitro. (a) The 12 h changes in Y74‐ADSCs cell proliferation with low glucose Dulbecco's Modified Eagle's medium (DMEM) after treated with different mitochondrial dosage (*n* = 6). (b) Cell counts and density change of groups in A, images taken by EOVS M7000 microscope. (c) Representative images of migration in mito‐transferred Y74‐ADSCs versus control Y74‐ADSCs (*n* = 3). Scale bars, 100 μm. (d) Percentage area of repaired wound. (e) Representative images of transwell migration assay in mito‐transferred ADSCs versus control ADSCs (*n* = 3). Scale bars, 50 μm. (f) Quantitative analysis of migrated cells by transwell assay. (g) Comparison of β‐galactosidase staining (blue), colony formation and multipotency of mito‐transferred Y74‐ADSCs versus control Y74‐ADSCs with and without doxorubicin‐induced oxidative stress. (h) ALP expression of representative cells in each group (*n* = 6). (i) Oil red O intensity of lipid droplets in each group (*n* = 6). (j) Optical density (blue) of Alcian blue staining (*n* = 5). Significantly different (one‐way analysis of variance [ANOVA]): ns, not significant, *p < 0.05, and ***p < 0.001

To confirm our main hypothesis of whether the proliferation of Y74‐ADSCs could be sufficiently enhanced by mitochondrial transfer, ADSCs were transfected with exogenous mitochondria at different doses for 6 h, followed by assessing cell proliferation for additional 12 h under serum starvation via CCK‐8 (Figure 3a). The proliferation rate was significantly higher in Y74‐ADSCs with exogenous mitochondria at 18.5 μg/ml than in nontreated Y74‐ADSCs served as control from 6 to12 h. At 12 h, the results of a cell counter showed that mito‐transferred ADSCs had up to twofold higher cell number compared to control ADSCs (Figure 3b). Increase cell density was also observed by phase‐contrast microscopy (Figure 3b), indicating that mitochondrial transfer has notable self‐renewal potential by ADSCs proliferation under serum starvation.

Subsequently, under the same condition‐serum starvation, we conducted both scratch wound healing and cell migration using live cell imaging. We first performed the scratch assay as wound healing is a critical step of tissue homoeostasis and repair. Y74‐ADSCs transferred with mitochondria displayed enhanced migration leading to accelerated gap closure of the cell‐free area compared with control ADSCs (Figure S5a,b). There was significantly greater healed area for mito‐transferred ADSCs (45.8% vs. 12.6% for control) (Figure 3c,d). Next, the results from transwell migration assays confirmed that mito‐transferred ADSCs were more migratory as they migrated through the porous membrane in increased cell numbers to the surface of the basolateral chamber (Figure 3e,f and Figure S6a,b). This indicates that exogenous mitochondria augment the mobility of ADSCs.

To further investigate the effect of mitochondrial transfer on motility, the migratory behavior of mito‐transferred ADSCs was analyzed by time‐lapse microscopy for 3 days. Without mitochondrial transfer, ADSCs were observed to migrate individually and slowly toward the central area of wound. When mitochondrial stimulus was present, ADSCs migrated collectively and resulting in a more rapid wound closure (Video S5). Therefore, these data suggest that mito‐transferred ADSCs are more resistant against the injury simulated by serum deprivation.

To elucidate the cellular response against ROS, the mito‐transferred ADSCs were exposed to Dox (200 nM) and examined for SA‐β‐Gal activity, colony‐formation ability, and multilineage differentiation potential. Prior to these experiments, we first checked the survival of ADSCs against Dox by measuring the changes in mitochondrial membrane potential using a JC‐1 assay. The monomeric JC‐1 displays green fluorescent and aggregates in a mitochondrial membrane potential‐dependent manner to display red fluorescence. Therefore, the color difference of the JC‐1 stained mitochondria can be used to assess the severity of mitochondrial damage caused by different treatments. As shown in Figure S7a,b, the addition of carbonyl cyanide 3‐chlorophenylhydrazone (CCCP) as negative control induced the complete loss of mitochondrial membrane potential in ADSCs as indicated by the JC‐1 green fluorescence, while Y74‐ADSCs without Dox as positive control exhibited strong magenta fluorescence. Y74‐ADSCs treated by Dox displayed yellow‐green fluorescence after 12 h of incubation, indicating partial depolarization of the mitochondrial membrane potential. Under the same condition, mito‐transferred ADSCs still showed strong magenta fluorescence, suggesting the unaltered mitochondrial membrane potential due to the incorporation of exogenous mitochondria. Cell death via apoptosis was further investigated by mitochondrial membrane potential/Annexin V assay. Flow cytometric analysis showed that the total ratio of dead/Annexin V positive cells in Y4‐ADSCs after incubation with Dox for 12 h was increased from 5% to 64%, while that in the mito‐transferred group was only 38% for ADSCs (Figure S7c). All these results demonstrate a superior survival rate due to mitochondrial transfer compared with conventional ADSCs.

Similarly, Dox‐treated Y74‐ADSCs showed an increase in the number of cells positive for SA‐β‐Gal activity as well as a decrease in number of colony‐forming units. In contrast, mito‐transferred Y74‐ADSCs has decreased the senescence marker levels and retained their colony forming ability to an extent comparable to healthy ADSCs (Figure 3g). In addition, staining for alkaline phosphatase (ALP), a key marker of osteogenesis, showed that the average cell number of ALP‐positive cells in Dox‐treated mito‐transferred Y74‐ADSCs was significantly higher than that in Dox‐treated Y74‐ADSCs but lower than nondrug‐treated cells (Figure 3g,h). Consistent with this finding, Alizarin Red staining revealed more substantial calcification in the mito‐transferred Y74‐ADSCs, as observed by mineralized nodules at Day 7 (Figure 3g). For the adipogenic potential, Oil red O stained images showed that lipid droplets were significantly accumulated in mito‐transferred Y74‐ADSCs, showing fivefold increase in the intensity compared with Y74‐ADSCs (Figure 3g,i). Lastly, stimulation of mito‐transferred Y74‐ADSCs after Dox treatment in chondrogenic culture resulted in intensification of Alcian blue staining when compared with Dox‐treated Y74‐ADSCs, suggesting an increased expression of anionic cell materials, such as chondroitin sulfate and keratan sulfate (Figure 3g,j). In other words, these results indicate that enhanced proliferation, migration, and differentiation shown by a number of in vitro assays are because of exogenous mitochondrial transfer.

### 2.4 Exogenous mitochondrial transfer modulates cell cycle progression and cellular secretomes

**FIGURE 4:**
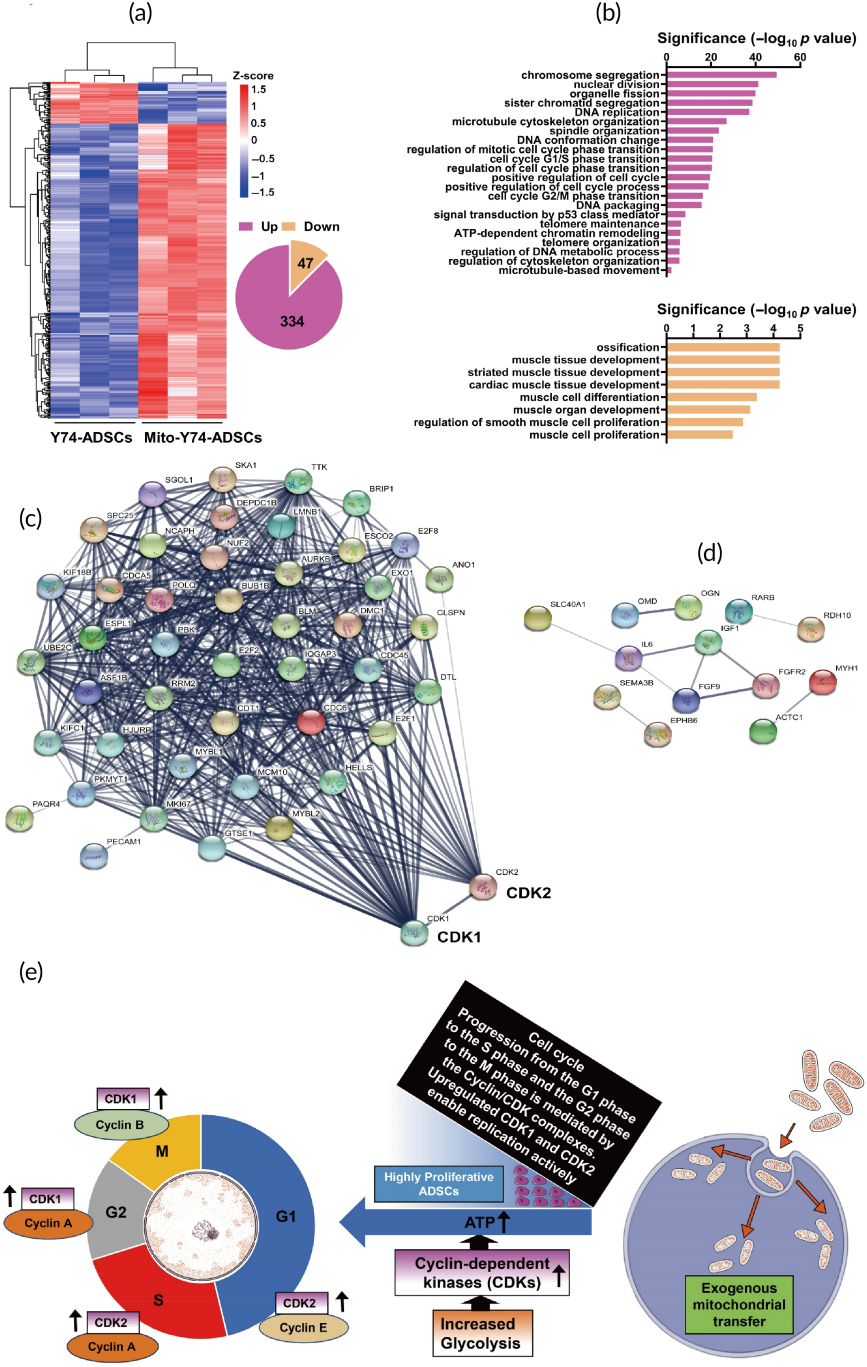
RNA‐seq analysis reveals differentially expressed genes in mito‐transferred Y74‐ADSCs. (a) Heat map of the 334 upregulated genes and the 47 downregulated genes in mito‐transferred Y74‐ADSCs versus control Y74‐ADSCs (*n* = 3). (b) Gene Ontology analysis of upregulated (top) and downregulated (bottom) by mitochondrial transplantation. (c) String analysis identified genes in mito‐transferred Y74‐ADSCs that associated with CDK1 and CDK2. (d) Gene networks displaying interactions between downregulated genes related to differentiation. (e) Schematic illustration of highly proliferative Y74‐ADSCs construction by incorporating exogenous mitochondria to accelerate the cell cycle. Exogenous mitochondrial transfer promotes the glycolysis, that efficiently produces ATP and stimulates recipient cell proliferation via enhanced CDK1/2. The activities of these CDKs are primarily regulated by the periodic expression of their cyclin‐binding partners, temporally control sequential cell cycle transitions through G1 to S phase and G2 to M phase. The division cycle contains long growth phase (G1) followed by a DNA synthesis phase (S) that is followed by a short growth phase (G2) before the next round of mitotic division (M)

To elucidate Y74‐ADSCs responses to mitochondrial transfer on the transcriptomic scale, we performed whole transcriptome RNA sequencing to analyze differential gene expression after 24 hpostmitochondrial transfer between nontreated and mito‐transferred cells. We found 334 genes being upregulated and 47 genes being downregulated (log_2_FC > 2, <−2, and *p* < 0.05) in mito‐transferred ADSCs compared with nontreated cells (Figure 4a). Gene Ontology (GO) analysis illustrated that genes highly expressed in mito‐transferred group are enriched in nuclear division, DNA replication, chromosome segregation, organelle fission, and microtubule cytoskeleton organization, implying such internalization of exogenous mitochondria may be advantageous in supplying additional energy during cytokinesis (Figure 4b). Most of the downregulated genes were associated with differentiation, such as osteogenesis, tissue development, and muscle cell differentiation, suggesting that mitochondrial simulation did not alter the retention of ADSC differentiation state (i.e., “stemness”).

Moreover, we established interaction networks of modulated genes in mito‐transferred ADSCs using the STRING database (https://string-db.org/). A co‐expression network was constructed for the top 46 node genes being upregulated (Figure 4c). Network analysis highlighted the role of cyclin‐dependent kinase (CDK) family in module formation, which were key control points as shared links among interacting genes. These cell cycle regulators may phosphorylate additional cell cycle related substrates in stem cell, which could further contribute to the rapid proliferation and maintenance of pluripotency. The reduction of gene expression involved in skeletal muscle differentiation (e.g., IL6, IGF1, OGN, and MYH1) also reflected mitochondrial delivery did not trigger undesirable differentiation in vitro (Figure 4d).

Collectively, we illustrated the underlying mechanisms according to RNA sequencing results (Figure 4e). Exogenous mitochondrial transfer promotes the glycolysis, that efficiently produces ATP and stimulates recipient cell proliferation via CDK1/2.

**FIGURE 5:**
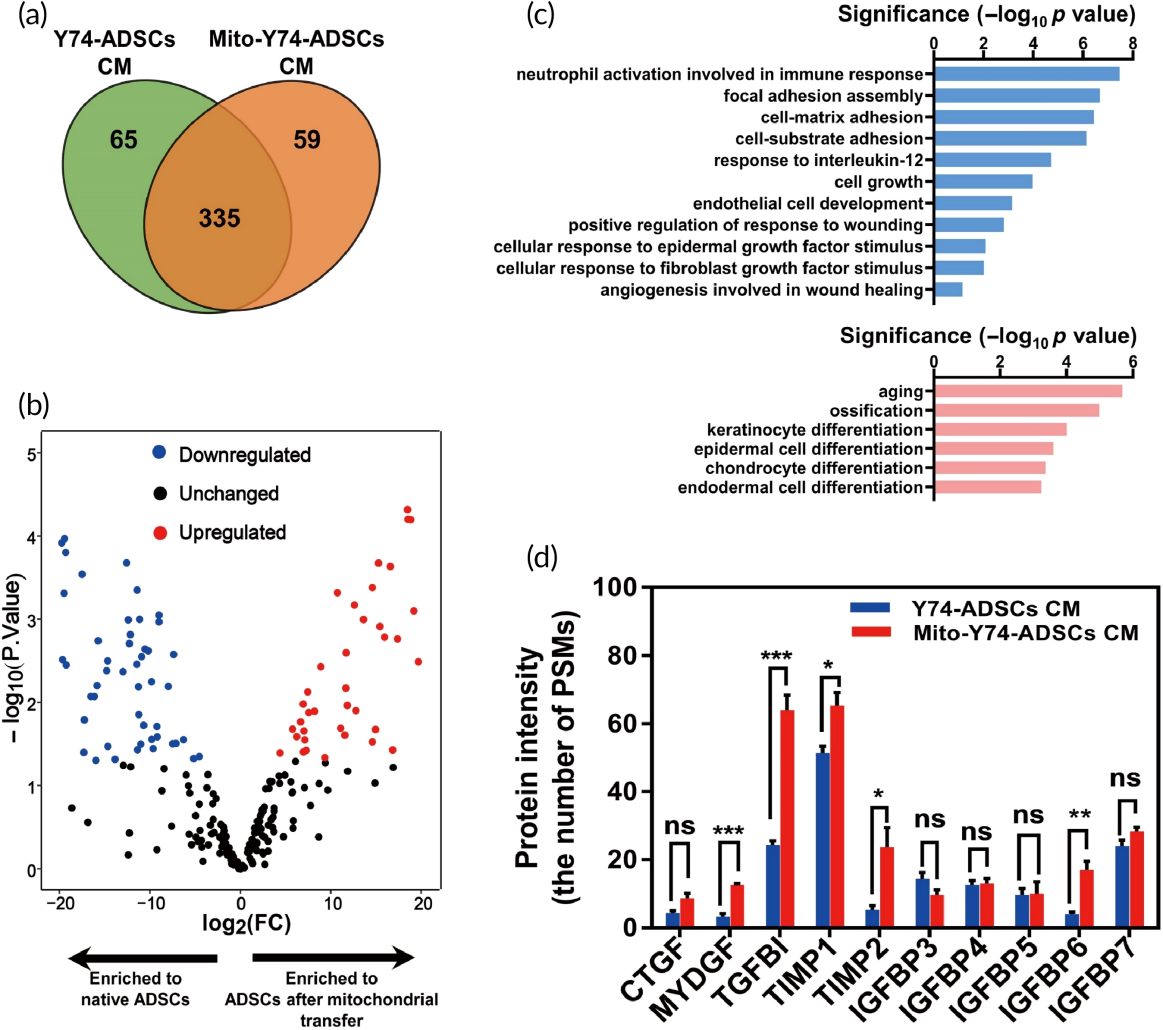
Cellular secretome changes that occur with mitochondrial transfer. (a) Venn diagram depicting unique and shared proteins in conditioned media (CM) derived from mito‐transferred Y74‐ADSCs versus control Y74‐ADSCs (*n* = 3). (b) Volcano plot indicating protein distribution in CM after mitochondrial transfer, as determined by quantitative proteomics. Positive (red; upregulated) and negative (blue; downregulated) correlations showed the log_2_ fold expression changes (log_2_FC > 2, <−2, and *p* < 0.05). (c) Gene Ontology analysis of functional annotations upregulated (top) and downregulated (bottom) by mitochondrial transplantation. (D) Several proteins involved in “immune response” and “cell growth” secreted in significantly different quantities by mito‐transferred Y74‐ADSCs versus control Y74‐ADSCs. Data are presented as the average number of PSMs. Significantly different (one‐way analysis of variance [ANOVA]): ns, not significant, *p < 0.05, **p < 0.01, and ***p < 0.001. CTGF, connective tissue growth factor; MYDGF, myeloid‐derived growth factor; TGFBI, transforming growth factor beta induced; TIMP1 and 2, tissue inhibitor matrix metalloproteinase 1 and 2; IGFBP3‐7, insulin‐like growth factor binding proteins family 3‐7

We next focused on the extracellular signals secreted by ADSCs after mitochondrial transfer. Therefore, we performed an in‐depth quantitative analysis of ADSCs derived paracrine factors in conditioned medium (CM) by LC/MS‐MS. Only proteins with at least two confidently identified peptides were included in the analysis. ADSCs shared a high degree of similarity before/after mitochondrial transfer, with 335 proteins identified to be unchanged. Fifty‐eight were expressed highly in the mito‐transferred Y74‐ADSCs CM (Table S1), whereas 64 were highly expressed in the control Y74‐ADSCs CM (Figure 5a). Volcano plot demonstrated the differential protein expression profile between mito‐transferred groups and controls (Figure 5b). GO annotation revealed the functions of upregulated and downregulated proteins with >2‐fold changes. Interestingly, biological processes including neutrophil activation involved in immune response, response to interleukin‐12, cell growth, positive regulation of response to wounding, angiogenesis involved in wound healing were enriched in the proteome of mito‐transferred ADSCs, which are of particular interest for therapeutic utility (Figure 5c). Consistent with our RNA sequencing data, the downregulated proteins were significantly enriched in categories related to differentiation. Based on these findings, we were especially interested in proteins involved in the activation of immunosuppression and the stimulation of cell growth and further extracted a set of secreted factors from the GO terms. Among them, we observed a significantly increased expression of TGF‐β targets, such as transforming growth factor beta induced (TGFBI) and insulin‐like growth factor binding proteins 6 (IGFBP6) (Figure 5d), which emerged as the key upstream regulator critical for enhanced immune response in mito‐transferred ADSCs. While secreted myeloid‐derived growth factor (MDGF) and tissue inhibitor matrix metalloproteinase 1 and 2 (TIMP1 and 2) were responsible for cell growth‐stimulating activity (Figure 5d). Comparison of proteomic profiles in mito‐transferred ADSCs and nontreated ADSCs secretome revealed significant differences in key signaling pathways and regulators involved in functionality by mitochondrial transfer.

### 2.5 Rapid tissue repair by transplantation of mito‐transferred ADSCs

**FIGURE 6:**
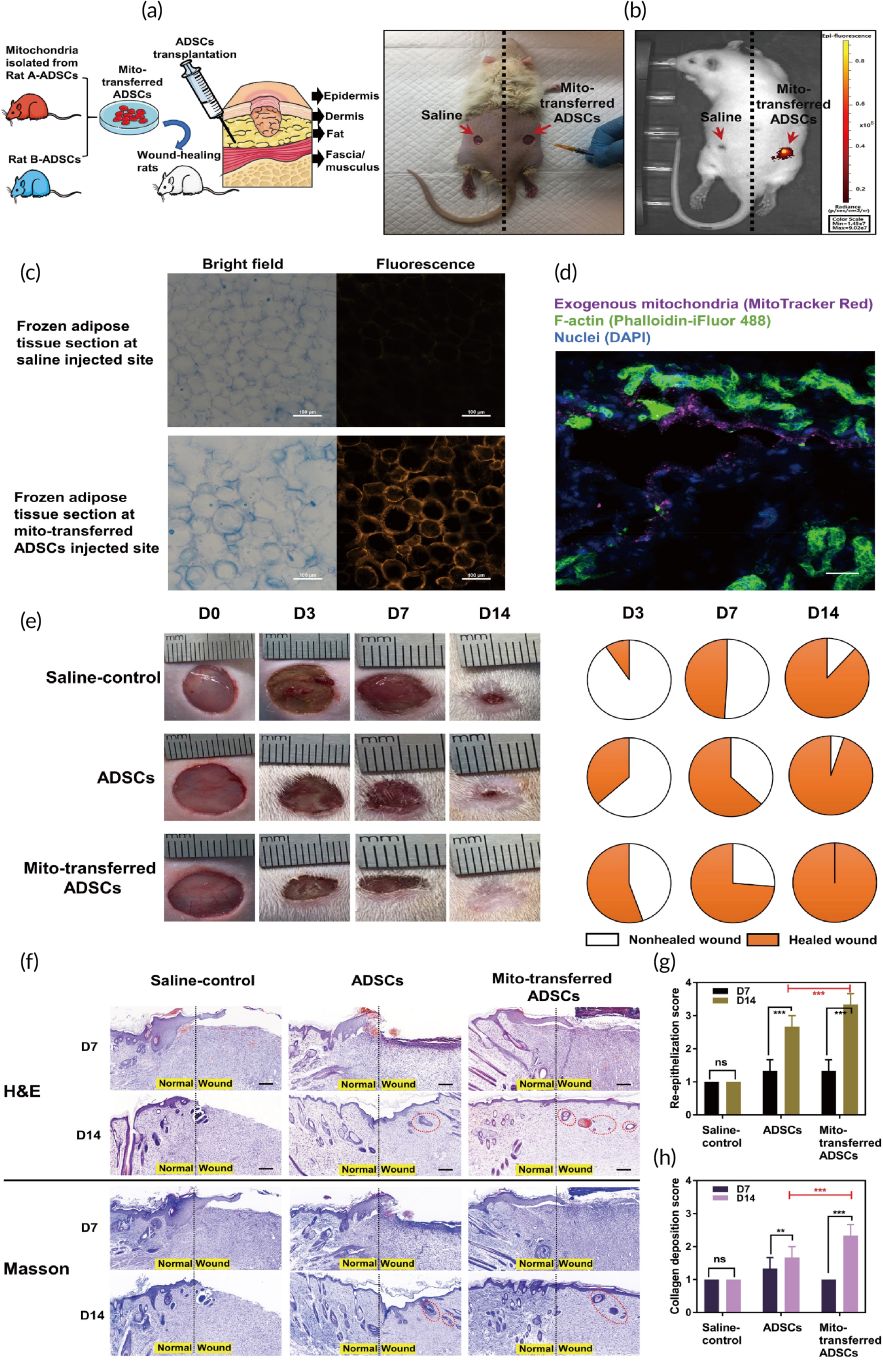
In vivo assessment of mito‐transferred ADSCs in a rat full‐thickness skin wound model. (a) Schematic illustration of in vivo ADSCs injection into the fat layer of each wound. Each wound had about 10 mm in diameter and was 2 mm deep. The experimental groups are (i) sham control, (ii) rat B‐ADSCs, and (iii) mito‐transferred rat B‐ADSCs. (b) Tracking of infused mito‐transferred ADSCs by in vivo imaging. (c) The presence of fluorescence labeled exogenous mitochondria in adipose tissue. (d) Representative immunostaining images of adipocytes with exogenous mitochondria (MitoTracker Red), F‐actin (Phalloidin‐iFluor 488) and DAPI for nucleus. Scale bars, 20 μm. (e) Representative images of the wound area (left) and the corresponding fractions of wounds healed (right) by different treatments on Days 0, 3, 7, and 14 after operation (*n* = 8). (f) H&E and Masson staining of the wound area reflected the regenerated skin in different treatments at Days 7 and 12 (*n* = 3). Scale bars, 100 μm. New hair follicles were highlighted by red cycles. (g,h) The functional scores of re‐epithelization and collagen deposition was scored 0 to 4 (*n* = 8). Significantly different (one‐way ANOVA): ns, not significant, **p < 0.01, and ***p < 0.001

Promoted proliferation and migration functions of mito‐transferred ADSCs in vitro could translate to a rapid tissue repair response in vi*vo*. The therapeutic effects were verified in a rat full‐thickness skin wound model (Figure 6a). Similar to mito‐transferred Y74‐ADSCs, mito‐transferred rat‐ADSCs were engineered by delivery of exogenous mitochondria isolated from Rat A‐ADSCs into ADSCs derived from Rat B using the serum starvation combined with co‐culture method. We included the following experimental groups: (i) saline‐control, (ii) rat B‐ADSCs, and (iii) mito‐transferred rat B‐ADSCs, followed by implanting total 6 × 10^6^ cells to the skin fat layer by direct injection. The presence of mito‐transferred ADSCs at the wound site after 6 h post‐transplantation was confirmed by labeling delivered mitochondria with MitoTracker Red CMXRos (Figure 6b). We further hypothesized that ADSCs might conversely donate mitochondria to the surrounding cells in the vivo environment. The fat tissue frozen sections showed Rat A‐ADSCs associated mitochondria‐positive adipocytes were detected in transplantation area (Figure 6c), and strong mitochondrial red fluorescent signal was confirmed by confocal imaging at 6 h post‐transplantation (Figure 6d), suggesting that the therapeutic benefit of mito‐transferred ADSCs not only offers advantages to stem cell themselves but may act as “mitochondrial delivery vehicles” to affect neighboring cells.

Wound size measurements demonstrated that saline‐ and control ADSCs‐treated wounds were slow for closure even after 14 days but the wound sites by mito‐transferred ADSCs were healed in 14 days (Figure 6e and Figure S8). Mito‐transferred ADSCs significantly accelerated wound closure, compared with saline control and nontreated ADSCs, with 60% closure at Day 3 relative to 10% for control and 30% for nontreated ADSCs; 100% of the wound area was closed by Day 14 with mito‐transferred ADSCs. Wounds with the various treatments were collected on Days 7 and 14 and stained with Masson's trichrome and hematoxylin and eosin (H&E) for histological analysis (Figure 6f). All treatments on Day 14 presented similar levels of inflammation with no severe immune response. However, saline‐control treated wounds had remaining gaps, while both control ADSCs and mito‐transferred ADSCs treated wound demonstrated a multilayered epithelial structure that firmly resembled native epidermis of the intact skin (Figure 6g). Moreover, Masson's trichrome and H&E staining demonstrated mito‐transferred ADSCs transplantation group resulted in more collagen distribution compared with control ADSCs (Figure 6h). On this basis, these results support the idea that ADSCs engineered by exogenous mitochondria enabled faster restitution of damaged tissues both at the morphological and functional level.

## 3 DISCUSSION

Despite the cognitive dissonance between preclinical findings in animal systems and human MSC clinical trial outcomes, MSCs are still the most frequently used cell source for tissue repair and regeneration due to their engrafting ability and broad differentiation potential.^27^ Thus, especially in the field of somatic stem cells, the focus has been on developing effective methods to maximize clinical potency of MSCs.^28^ In this study, we have introduced a novel alternative bioengineering approach to potentiate the therapeutic efficacy of MSCs through transfer of exogeneous mitochondria. Intracellular mitochondrial transfer modified cellular energy metabolism, which can effectively boost the innate functions of MSCs, allowing for improved in vivo tissue regenerations.

Genetically engineered MSCs (either viruses or noninsertional gene transfer) are usually designed to secrete highly expressed various biologicals targeted against specific disease. The clinical use of these is restricted by packaging problems and safety concerns.^29^, ^30^ In comparison, mitochondria are naturally occurring particles with low immunogenicity. Using serum starvation combined with co‐culture system, we assemble direct evidence for an easy‐to‐perform, robust, and amenable to clinical‐scale expansion of MSCs transferred with isolated mitochondria from healthy donor stem cells (Figure 1). To the best of our knowledge, this is the first study to follow in vitro acquisition of exogenous mitochondria in real time, and we optimize the effective mitochondrial dose (Figure 2 and [Link], [Link]). The endocytic existence of active and functional exogenous mitochondria was visualized and quantified using MitoTracker Red CMXRos dye, which accumulates in active mitochondria with an intact mitochondrial membrane potential.^31^, ^32^ With a mitochondrial dose of 18.5 μg/ml per 10^4^ ADSCs, mito‐transferred ADSCS displayed a 17% increase in ATP production. These cells retained high multipotency, which is a hallmark of “super” stem cells.

The energy metabolism of undifferentiated MSCs has been reported to have more glycolysis dependence under aerobic conditions compared with OXPHOS dependence.^33^ This phenomenon is called as the “Warburg effect,” that operates predominantly in highly proliferative cells.^34^ Either energy or various types of cellular constituents, such as amino acids, lipids, or nucleotides, are essential for cell division. Indeed, glycolysis along with the pentose phosphate pathway could explain cellular constituents as well as ATP.^35^ Therefore, glycolysis is expected to be benefit for self‐renewal of the actively proliferating stem cells and “stemness” maintenance. Glycolytic metabolism produces fewer ROS in cells.^36^ Interestingly, the elevation in the nonmitochondrial oxygen consumption was detected in mito‐transferred ADSCs (Fig. 2h). Several studies suggested that high nonmitochondrial respiration promoted oxidative stress followed by increasing ROS generation, which is an indicator of impaired mitochondrial function.^37^, ^38^ Conversely, other researches evidenced that increased nonmitochondrial oxygen consumption was not necessarily associated with ROS, and this phenomenon could be due to the processes including protein folding, synthesis of lipid and collagen, demethylation, and hydroxylation, because mitochondrial oxidative phosphorylation is not the unique way to consume oxygen in cells.^39^ To confirm this, we performed DNA damage analysis using 8‐OHdG level detection in mito‐transferred ADSCs (Figure 4s), showing there was no existence of oxidative stresses after exogenous mitochondrial incorporation. Based on our observations (Figure 2i), the glycolytic rate assay delineated the sole contribution of glycolysis to the enhanced ATP production rate of mito‐transferred ADSCs. We infer that the exogenous mitochondria may induce a metabolic switch from OXPHOS to glycolysis in the bioengineered stem cells, which is similar to the shift during induced pluripotent stem cell (iPSC) reprogramming.

To further encompass these bioenergetics consequences related to mitochondrial uptake, transcriptome analysis was performed on mito‐transferred ADSCs populations. Bioinformatic analysis demonstrated that the most significant alterations in mRNA expression levels were found in cell cycle, particularly CDK1 and CDK2 (Figure 4c). The activities of these CDKs are primarily regulated by the periodic expression of their cyclin binding partners, temporally control sequential cell cycle transitions through G1/S phase and G2/M phase.^40^ Stimulation of cells with hormones, growth factors, microRNAs, or small molecules induces expression of CDKs and cyclins and accelerates cell cycle progression.^41^ At the mechanistic level, it is well known that CDKs contribute to the promoted self‐renewal by activating the PI3K‐Akt pathway.^42^, ^43^ Notably, the knockdown of CDK1, CDK2 or treatment with CDK‐inhibitors was reported to trigger differentiation in stem cells,^44^ whereas overexpression of CDKs coordinately controls cell proliferation and migration.^45^, ^46^ Our observation corroborates these findings, with mito‐transferred ADSCs showing greater proliferation, migration, and multilineage differentiation compared with control ADSCs (Figure 3). In addition to our transcriptomic data, secretome analysis also sheds light on the molecular changes associated with mito‐transfer, including cytokine levels and anti‐inflammatory potential (Figure 5). We identified TGFBI and TIMP 1 to be highly represented in the conditioned medium of mito‐transferred ADSCs. TGFBI is an extracellular matrix protein that known to modulate homeostasis by promoting cell adhesion and microtubule stabilization.^47^ TIMP 1 is a metalloproteinases inhibitor reported to have an antiapoptotic effect.^48^ This implies that mitochondrial transfer may improve the secretome of ADSCs to enhance cell therapy.

**FIGURE 7:**
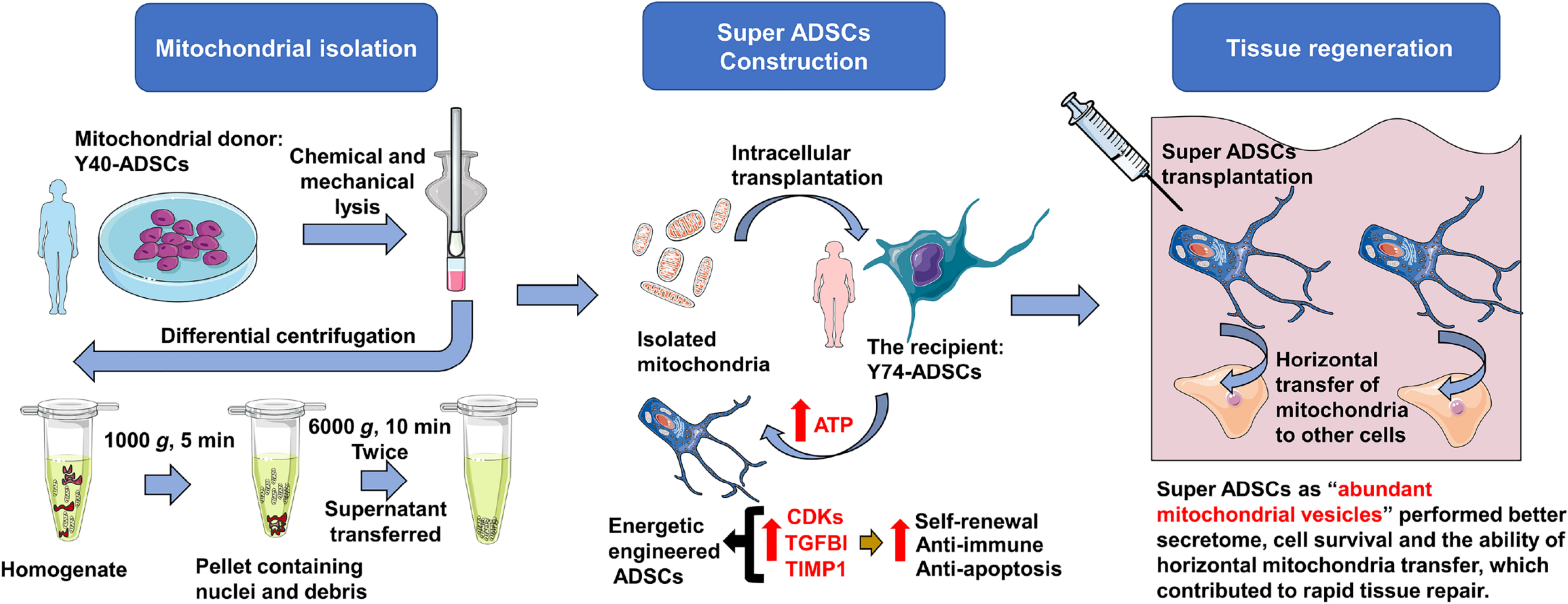
Schematic illustration of the super adipose‐derived mesenchymal stem cells (ADSCs) construction by intracellular mitochondrial allotransplantation for tissue repair. Donor mitochondria are isolated and purified from donor cells (Y40‐ADSCs) by differential centrifugation, followed by uptake into the recipient ADSCs via serum starvation. This results in “super ADSCs” with the aim to optimize stem cell therapy in vivo

The substantial cell‐to‐cell variation is widely shown between MSCs. Such heterogeneity develops further during in vitro culture and population expansion. Such pervasive variability also limits their therapeutic efficacy.^49^, ^50^ Our results indicated that intracellular mitochondrial transfer results in highly homogeneous ADSCs for a rapid in vitro healing (Video S5), which was subsequently observed in the in vivo setting. Interestingly, we unexpectedly found evidence that the engineered ADSC may also act as “carriers” to share mitochondria with surrounding cells after injection (Figure 6c,d). It is yet unclear whether those exogenous mitochondria are released from the ADSCs by the injected fluid pressure instead of “ADCS mediated horizontal transfer”. Some mitochondrial transplantation studies claim that if exposed to in vivo calcium at millimolar concentrations, isolated mitochondria failed to oxidize pyruvate and malate irreversibly, without any ATP formation because of the opening of the mitochondrial permeability transition pore.^51^, ^52^ In other words, isolated mitochondria without shielding are less likely to survive by direct transplantation. In our case, despite dermal calcium gradients varying from 0.5 mM to > 1.4 mM,^53^ the fluorescent membrane potential staining of exogenous mitochondria was still observed after 6 h post‐transplantation (Figure 6b), suggesting the stem cells packed with exogenous mitochondria can be used as a “Trojan‐horse” delivery method to preserve their activities rather than direct isolated mitochondrial injection. Benefiting from those exogenous mitochondria, we propose that mito‐transferred ADSCs display improved survival, increased amounts of beneficial paracrine factors and the capacity of secondary mitochondrial transfer and consequently accelerate wound closure (Figure 7). Future directions related to this work will focus on cellular and molecular mechanism level of tissue repair mediated by mito‐transferred ADSCs transplantation.

The study demonstrated the potential feasibility for constructing energetically engineered stem cells by transfer of nonautologous mitochondria from healthy donor ADSCs into normal recipient ADSCs. Both the plasticity and paracrine secretion are significantly up‐regulated by enhanced bioenergetics. From a clinical perspective, these “supercharged” stem cells may ultimately improve the efficacy of regenerative medicine.

## 4 EXPERIMENTAL SECTIONS

### 4.1 Isolation, expansion, and characterization of human ADSCs

Human ADSCs were isolated from the liposuction specimens of healthy female donors aged 40 and 74 years, respectively, who underwent liposuction surgery (abbreviated as Y40‐ADSCs and Y74‐ADSCs). The Institutional Review Board (IRB) of The First Affiliated hospital of Zhejiang University School of Medicine (IRB no. 2018‐392) approved the application of these human sourced cells. The isolation procedure was described previously.^54^ Adipose tissue samples were rinsed extensively with Hank's balanced salt solution (HBSS) (Cat. No. 14025092, Gibco) to eliminate blood cells, followed by soaking into collagenase type I (0.1%, v/v; Cat. No. 17100017, Gibco) at 37°C until over 95% tissue was digested. After centrifugation at 1500 rpm for 10 min, the pellets were filtered through a 100‐μm strainer. The cell suspension solutions were further centrifugation at 1500 rpm for 10 min and resuspended in Dulbecco's Modified Eagle's medium low‐glucose (DMEM) (Cat. No. 11885084Gibco) with fetal bovine serum (FBS) (10%, v/v; Cat. No. 10099141C, 2206991CP, Gibco) and penicillin streptomycin solution (P/S) (1%, v/v; Cat. No. 15070063, Gibco) to generate primary ADSC cultures.

During culture, adherent cells were trypsinized from a plastic culture plate surface, and their immunophenotype was confirmed by flow cytometry (NovoCyte; ACEA) using the following monoclonal antibodies: CD73‐PE (Cat. No. 344004, Biolegend), CD90‐PE (Cat. No. 555596, Biosciences), CD105‐PE (Cat. No. 12‐1057‐42, Ebioscience), CD34‐PE (Cat. No. 343606, Biolegend), and CD45‐PE (Cat. No. 368510, Biolegend). For staining, all cells were rinsed by staining/wash buffer with 30 min incubation at 4°C avoiding light.

A luciferase‐based ATP Assay Kit (Cat. No. S0027, Beyotime) was used for evaluating the total amount of ATP produced from Y40‐ADSCs and Y74‐ADSCs with a population of 1 × 10^6^ cells. We followed the manufacturer's protocol.

To quantify the mitochondrial amount, Y40‐ADSCs and Y74‐ADSCs genomic DNA (500‐1000 ng) were purified using NucleoSpin Tissue (Cat. No. 740952.50, Takara Bio) followed by RT‐PCR (Bio‐rad) to calculate their relative number of copies of mtDNA using Human MtDNA Monitoring Primer Set (Cat. No. 7246, Takara Bio). Triplicate qRT‐PCR reactions were run in three independent experiments. The copy number of mtDNA is calculated in terms of the average of the values calculated by the primers combinations as the ND1/SLCO2B1 pair and the ND5/SERPINA1 pair as given in the product manual.

Mitochondrial morphology of Y40‐ADSCs and Y74‐ADSCs was characterized by TEM.^55^ Briefly, cells were fixed for 2 h by 2.5% glutaraldehyde in 0.2  M sodium cacodylate buffer (pH 7.2), with a 1 h secondary fixation by 1% osmium tetroxide in 0.2 M sodium cacodylate buffer (pH 7.2). After rinsed three times with water, the cells were further fixed and stained by 2% aqueous uranyl acetate for 30 min and dehydrated in a graded ethanol (50%, 70%, 90%, and 100% of ethanol, each for 15 min), infiltrated with a 1:1 Epon/Acetone mixture and imbedded in a 100% Epon. Thin sections were stained with lead citrate and visualized by using HT7700 TEM (Hitachi).

Mitochondrial density and distribution were further visualized by Mitotracker fluorescence labeling captured by confocal microscopy with 10x objective (LSM 880; Zeiss). For donor Y40‐ADSCs, nuclei were labeled with Hoechst 33342 (Cat. No. H3570, Thermo Scientific) (Blue) (DAPI channel) and mitochondria were treated with 200 nM MitoTracker Red CMXRos (Cat. No. M7512, Invitrogen) (Texas Red channel) for 45 min at 37°C and rinsed three times with DMEM for live‐cell imaging. In contrast, for recipient Y74‐ADSCs, mitochondria staining used 200 nM MitoTracker Green FM (Cat. No. M7514, Invitrogen) (FITC channel) instead. This visual discrimination was used for all the experimental procedures unless stated otherwise.

### 4.2 Isolation of mitochondria from donor Y40‐ADSCs

To isolate sufficient quantity of mitochondria, 15 cm cell culture dishes were used for Y40‐ADSCs amplification. The cells were collected when they achieved 90% confluence and were re‐seeded with a density of 3 × 10^5^ cells/cm^2^. The cells were amplified two to four passages in a GMP (Good Manufacturing Practice)‐compliant facility. The cells were stained before mitochondrial isolation. Donor Y40‐ADSCs were rinsed with DMEM. Then, the cells were incubated in dye solution containing 200 nM MitoTracker Red CMXRos in DMEM for 45 min. The cells were rinsed three times with PBS after staining, followed by trypsinization and centrifugation at 600*g* for 5 min. The collected pellets were then used for mitochondrial isolation.

The isolation of mitochondria was achieved using Qproteome mitochondria isolation kit (Cat. No. 37612, Qiagen) combined with Potter‐Elvehjem homogenizer (3 ml capacity). The procedures for cell lysis and subsequent centrifugations followed the modified protocols as below. Step1: Cell pellets were suspended into 1 ml of ice‐cold lysis buffer (Qiagen) for 5 min incubation followed by centrifugation at 1000*g* for 5 min at 4°C. Step 2: The precipitated cell pellets were then resuspended in a Potter‐Elvehjem homogenizer with 1 ml ice‐cold disruption buffer (Qiagen) and disrupted cells by moving the pestle up and down for 30 times in the ice bath. Step 3: The large cell debris were separated by centrifugation at 1000*g* for 5 min at 4°C. Step 4: The supernatant was further centrifuged at 6000*g* for 10 min at 4°C to collect the mitochondrial pellets. Step 5: The mitochondrial pellets were rinsed with 1ml mitochondria storage buffer (Qiagen) and isolated by another centrifugation at 6000*g* for 10 min at 4°C. Isolated mitochondria were applied freshly for the subsequent characterizations.

The disruption efficiency at Step 2 was checked using flow cytometer (NovoCyte; ACEA) by Hoechst 33342 and living cell staining MitoTracker Red CMXRos. Intact cells population showed positive Hoechst 33342 and high MitoTracker Red CMXRos, while the lysates gave negative Hoechst 33342 and high MitoTracker Red CMXRos. Data are shown as dot‐plot for average signal intensity of 100,000 ungated events.

Purify of mitochondria at Step 5 was evaluated by Western Blotting. Briefly, 4x SDS sample buffer was added to isolated mitochondria and cytosol fractionation, and then heated to 98°C for 5 min. Samples and markers were loaded onto SurePAGE gel (Cat. No. M00653, GeneScript) and transferred to PVDF membrane (Sigma‐Aldrich). Subsequently, the membrane was blocked with 1× TBST (Tris‐buffered saline with Tween 20) containing 5% nonfat dry milk, followed by incubating with primary antibody (GLS, Cat. No. ab156876, Abcam; rabbit‐anti TOMM20, Cat. No. 42406, Cell Signaling Technology; RPL9, Cat. No. ab182556, Abcam; β‐actin, Cat. No. A00702, GeneScript) overnight at 4°C. The membrane was rinsed three times with 1× TBST for 10 min each. The membrane was incubated with a suitable secondary antibody (Goat anti‐Rabbit A32735, Goat anti‐mouse A32729, Thermo Fisher). After another three washing with TBST, the membrane was imaged using an Odyssey Fc Imaging system (LI‐COR).

### 4.3 Characterization of isolated Y40‐ADSCs mitochondria

To examine the structural integrity, isolated mitochondria were resuspended in mitochondria storage buffer (Qiagen) and mounted on a microscope glass slide to be immediately visualized by the microscope Leica DMI8 with 40× objective. MitoTracker Red CMXRos accumulates in active mitochondria in response to high mitochondrial membrane potential. Isolated mitochondria maintaining red fluorescence therefore indicates its viability.

The mean size distribution and zeta potential of isolated mitochondria were evaluated by DLS (Nano ZS, Malvern; 173° scattering angle at 25°C; 1.40 refractive index). The measurements were completed following appropriate dilution of the mitochondrial particles in storage buffer. Yield of mitochondria extracted from Y40‐ADSCs was calculated by the following equation: Yield (%) = *W*_mito_ */W*_cel_ × 100%, where *W*_mito_ is the weight of isolated mitochondrial pellets and *W*_cell_ is the weight of total cells before mitochondrial isolation.

ATP levels of isolated Y40‐ADSCs mitochondria were measured by the ATP Determination Kit (Cat. No. A22066, Invitrogen) according to manufacturer's protocol.^56^ Luminescence was measured by Spark 20M multimode microplate reader (Tecan). For normalization, mitochondrial protein was measured by bicinchoninic acid (BCA) assay (Cat. No. 23225, Thermo Scientific). For mitochondrial DNA quantitation, isolated mitochondria were required to be lysed in mitochondrial lysis buffer (Cat. No. C3601‐4, Beyotime) followed by lyophilization (50°C for 60 min) and ethanol precipitation (−20°C for 10 min) to obtain DNA. By checking the purity at OD260/OD280 ratio ≥1.8, total mitochondrial DNA was measured using a NanoDrop One Microvolume UV‐Vis Spectrophotometers (Thermo Scientific).

### 4.4 Generation of mitochondria transferred Y74‐ADSCs

The recipient Y74‐ADSCs (1 × 10^4^) were seeded onto a 96‐well plate and cultured in low‐glucose DMEM (Gibco) with FBS (10%, v/v; Gibco) and P/S (1%, v/v; Gibco) of 3 h for adherence at 37°C with 5% CO_2_. Subsequently, serum‐free low‐glucose DMEM was replaced for another 2 h incubation to enhance mitochondrial uptake efficiency. After the removal of medium and washing with PBS, fresh isolated mitochondria with different concentrations (1.85–18.5 μg/ml) were plated on top of Y74‐ADSCs with Minimum Essential Medium (MEM) without calcium for up to 12 h co‐incubation at 37°C with 5% CO_2_. After that, any mitochondria not transferred into cells were removed. Finally, the resultant cells were cultured in serum‐containing medium of 12 h for recovery. Optimization of these bioengineering parameters is further detailed on the following high‐content analysis. A recent study pointed out that bioenergetics of recipient cells after mitochondrial transplantation significantly decreased with increasing passage number and returned to physiological levels.^57^ For this reason, in our study, mitochondria transferred Y74‐ADSCs were used for direct characterizations and therapeutics without further expansion.

### 4.5 High‐content imaging and analysis

Nonautologous mitochondria isolated from donor Y40‐ADSCs were stained with MitoTracker Red CMXRos (excitation/emission 579/599 nm). Y74‐ADSCs derived mitochondria were labeled by MitoTracker Green FM (excitation/emission 490/516 nm). The nuclei of Y74‐ADSCs were labeled with Hoechst 33342 (excitation/emission 350/461 nm). Live cell tracking of the dynamic behavior of mitochondrial internalization was performed on the IN Cell Analyzer 2500HS (GE) with a 20x/0.75 Plan Apo objective (Nikon) under the controlled atmosphere (5% CO_2_ and 20% O_2_) and constant temperature at 37°C. Images were acquired every 15 min intervals for 12 h incubation with 100 ms exposure. Twenty FOVs with fixed spacing were captured per well, and each FOV contained approximately 290 cells. Each treatment was analyzed with eight repeated wells.

For quantitative analysis, image segmentation was performed by the GE Developer software.^58^ Briefly, cells were segmented using the “find nuclei” by the Hoechst blue channel and “find cytoplasm” by MitoTracker Green FM channel. To exclude apoptotic or dividing cells, intensity, and morphological measurements were picked up from the segmented cells. MitoTracker Red CMXRos‐positive cells were highlighted using the building block called “find spots.” For each FOV, the software returned the average of total intensity of MitoTracker Red CMXRos associated with the cell region represented as “the uptake of isolated mitochondria” and the average of total intensity of MitoTracker Green FM associated with the cell region represented as “the status of natural mitochondria.”

### 4.6 Metabolic flux assays

To investigate the bioenergetics consequences of mitochondria transferred ADSCs, the Cell Mito Stress Test (Cat. No. 103591‐100, Agilent) and Real‐Time ATP Rate Assay (Cat. No. 103592‐100, Agilent) were measured using a Seahorse XFp Analyzer according to the manufacturer's instructions with some modifications. Briefly, mitochondria transferred Y74‐ADSCs or control Y74‐ADSCs were seeded onto the miniplate (Agilent) with growth medium at the optimized density of 2 × 10^4^, respectively. To achieve an even distribution of cells within wells, the miniplates were rocked for 20 min at room temperature, subsequently cultured in a 37°C CO_2_ incubator for 2 h to allow cell adhesion. The growth medium was replaced with assay DMEM (Agilent) in time. The miniplates assembled with hydrated sensor cartridges were moved into a 37°C non‐CO_2_ incubator for 1 h prior to loading drug. Upon completion of the Seahorse assay, metabolic data were normalized by cell counts per well using EOVS M7000 microscope (Invitrogen) to ensure consistent interpretation of results.

In the Cell Mito Stress Test, sequential treatment with oligomycin (1.5 μM; Agilent) at Port A, FCCP (0.5 μM; Agilent) at Port B, and Rot/AA (0.5 μM; Agilent) at Port C was performed. To run the assays, the miniplate loaded with sensor cartridge was placed on the instrument tray and started with the corresponding programs. The Report Generator (Agilent) automatically calculated the Cell Mito Stress assay parameters (ATP production, the decrease in OCR following the injection of Oligomycin; nonmitochondrial respiration, minimum rate measurement after injection of Rotenone/antimycin A; basal respiration, last rate measurement before first injection – nonmitochondrial respiration; maximal respiration, maximum rate measurement after FCCP injection – nonmitochondrial respiration; and spare respiratory capacity, maximal respiration – basal respiration). In the Real‐Time ATP Rate assay, sequential treatment with oligomycin (1.5 μM; Agilent) at Port A and Rot/AA (0.5 μM; Agilent) at Port B were used. The XF Real‐Time ATP Rate assay parameters (mitoATP production rate, OCR_ATP_ (pmol O_2_/min) × 2 (pmol O/pmol O_2_) × P/O (pmol ATP/pmol O); glycoATP production rate, glycoPER [pmol H^+^/min]; and total ATP production rate, glycoATP production rate + mitoATP production rate) were calculated using the Report Generator and compared with that of the control group to evaluate the effectiveness of mitochondrial transfer by three sets of independent studies.

### 4.7 DNA damage assays

Commercially available 8‐hydoxy 2 deoxyguanosine (8‐OHdG) ELISA kit (Cat. No. ab201734, Abcam) was used to determine any potential oxidative DNA damage in mitochondrial transferred ADSCs in terms of the manufacturer's instructions. Briefly, the DNA of mitochondria transferred Y74‐ADSCs or control Y74‐ADSCs were purified by NucleoSpin Tissue (Cat. No. 740952.50, Takara Bio) followed by digesting using nuclease P1 (Cat. No. M0660S, NEB). After adjusting pH to 8 by 1 M Tris, alkaline phosphatase (one unit per 100 μg DNA) (Cat. No. D7027, Beyotime) was added for 10 min incubation at 37°C and deactivated at 75°C for another 5 min. Finally, 1.67 μg/well of ssDNA samples was performed by kit. Data were normalized to ng of 8‐OHdG per μg of DNA (*n* = 3).

### 4.8 Secretome analysis

A 1 × 10^6^ of mitochondria transferred Y74‐ADSCs or control Y74‐ADSCs was cultured in low‐glucose DMEM (Gibco) with FBS (10%, v/v; Gibco) and P/S (1%, v/v; Gibco) of 3 h for adherence at 37°C with 5% CO_2_, washed three times with PBS and starved in serum‐free DMEM for 24 h. The harvested conditioned media (CM) (*n* = 3 biological replicates for each cell type) was filtered using 0.2 μm filter (Millipore), concentrated using Amicon® Ultra‐15 Centrifugal filter with a 3‐kDA cutoff (Millipore) at 3900 *g* and 4°C. Three aliquots of each CM (2 ml) were stored at −80°C for further analysis. For mass spectrometry, 2 ml CM was dehydrated using Alpha 2‐4 LDplus (Christ) and reconstituted in 50 mM NH_4_HCO_3_. A 90 μg of protein samples was counted by BCA assay (Thermo Scientific) and reduced by 20 mM DTT before alkylated with 40 mM iodoacetamide. All crude protein extracts were precipitated by acetone at −20°C for 2 h, followed by dissolving in a triethylammonium bicarbonate (TEAB) buffer. Finally, trypsin was added (trypsin/protein, 1:50) and incubated at 37°C for 16 h. Digestion was stopped by the addition of 2% trifluoroacetic acid. After desalting with C18 solid‐phase cartridge, the tryptic digests were first separated on a U3000 HPLC System (Thermo Fisher Scientific, USA) using a BEH RP C18 column (5 μm, 300 Å, 250 mm × 4.6 mm i.d., Waters Corporation, USA). The gradient elution was composed of mobile phases A (ddH_2_O, adjusted pH to 10.0 using NH_3_·H_2_O) and B (98% acetonitrile, adjusted pH to 10.0 using NH_3_·H_2_O), with a setting as follows: 5%–8% B, 1 min; 8%–50% B, 6 min; 50%–80% B, 14 min; 80%–95% B, 1.5 min; 95% B, 6 min; 95%–5% B, 2 min. The separation was performed at an eluent flow rate of 1.0 ml/min with monitoring at 214 nm. The temperature of column oven was maintained at 45°C, and the eluent was collected every 90s. Ten fractions were collected for each sample. After vacuum drying and reconstitution in 20 μl of 0.1% (v/v) formic acid, 2% (v/v) acetonitrile in water, the samples were subsequently separated using a C18 column (75 μm inner diameter, 360 μm outer diameter × 15 cm, 2 μm C18). In terms of the hydrophobicity of fractions eluted in 1D LC, a series of adjusted linear gradients was employed with a flow rate of 300 nl/min, using mobile phase A (0.1% formic acid in water solution) and mobile phase B (0.1% formic acid in 80% acetonitrile solution). The MS conditions were given as follows: the electrospray voltage was 1.9 kV for Orbitrap Fusion Lumos, with no sheath gas flow and keeping the ion transfer tube at 350°C. In a data‐dependent mode, the survey scan of MS ranged from m/z 350 to 1500 with a resolution of 60,000 at m/z 200, while The MS2 spectra acquisition was performed at 15,000 resolution. Moreover, 20 of most intense peaks with charge state ≥2 were fragmented in collision‐induced dissociation with normalized collision energy of 30%, intensity threshold 1000, and one microscan. A tandem mass spectrum of specific fragment ions for each peptide was obtained after the detection, isolation, and fragmentation.

For quantitative analysis, we employed a LIMMA‐based analysis pipeline.^59^ Briefly, proteomics data were normalized using the *normalizeBetweenArrays* function and a linear model for each protein was fit via the *lmFit* function. Proteins with foldchange >2 and *p*‐value < 0.5 were defined as differentially expressed proteins. To reveal minor differences in secreted factors between mitochondria transferred Y74‐ADSCs and control Y74‐ADSCs from the GO terms, mass spectrometry‐based spectral count approach by the number of peptide spectrum matches (PSMs) was employed for each sample as an expression of its relative abundance. The sum of PSM in each sample (*n* = 3) was used to normalize the number of PSM.

### 4.9 RNA‐sequencing and bioinformatic analysis

A 1 × 10^6^ of mitochondria transferred Y74‐ADSCs or control Y74‐ADSCs (*n* = 3 biological replicates) was lysed to extract total RNA by TRIzol® Reagent following the manufacturer's instructions (Cat. No. 15596026, Invitrogen), with removal of genomic DNA by DNase I (Cat. No. 2270B, TaKara Bio). The RNA was qualified and quantified by 2100 Bioanalyser (Agilent) and the ND‐2000 (NanoDrop Technologies), respectively. Sequencing library was construct by high‐quality RNA samples (OD260/280 = 1.8–2.2, OD260/230 ≥ 2.0, RIN ≥ 6.5, 28S:18S ≥ 1.0, >10 μg).

RNA‐seq transcriptome libraries were built based on TruSeqTM RNA sample preparation Kit from Illumina (San Diego, CA) using 1 μg of total RNA. Briefly, messenger RNA obtained from polyA selection by oligo(dT) beads was fragmented using fragmentation buffer. By following Illumina's protocol, cDNA synthesis, end repair, A‐base addition and ligation of the Illumina‐indexed adaptors were completed. Next, size selection of cDNA target fragments of 200–300 bp on 2% low range ultra agarose was performed in the libraries, with 15 cycles of PCR amplification using Phusion DNA polymerase (NEB). After quantification using TBS380, Illumina NovaSeq 6000 sequencing (150 bp*2, Shanghai BIOZERON Co., Ltd) was used to sequence Paired‐end libraries.

Trimmomatic with parameters (SLIDINGWINDOW: 4:15 MINLEN:75) (version 0.36 http://www.usadellab.org/cms/uploads/supplementary/Trimmomatic) trimmed the raw paired end reads to enable the precision and control quality. Then, hisat2 (https://ccb.jhu.edu/software/hisat2/index.shtml) software used for mapping with default parameters allowed clean reads separately aligned to reference genome with orientation mode. The quality of these data was assessed by qualimap_v2.2.1 (http://qualimap.bioinfo.cipf.es/). Htseq‐count was used for each gene reads (https://htseq.readthedocs.io/en/release_0.11.1/).

DESeq2 was applied to identify differential expression genes (DEGs).^60^ The DEGs between two samples were chosen using the criteria as below: the logarithmic of fold change was >2 and the false discovery rate (FDR) was <0.05. To explore the functions of the differentially expressed genes, GO functional enrichment analysis was done by clusterProfiler.^61^ GO terms and metabolic pathways at Bonferroni‐corrected *p*‐value < 0.05 were considered as significantly enriched among DEGs. To further analyze the biological impact of DEGs, protein–protein interaction networks were created using the STRING database.

### 4.10 In vitro characterization of mitochondria transferred Y74‐ADSCs

The conventional and mitochondria transferred Y74‐ADSCs were seeded at density of 2000 cells/well under serum starvation conditions, respectively. The proliferation was evaluated by the Cell Counting Kit‐8 (CCK‐8) (Cat. No. 96992, Sigma‐Aldrich). The 450 nm absorption value was recorded at 0, 2, 4, 6, and 12 h, respectively, using a microplate reader (Tecan). The well scanning was run by EOVS M7000 microscope (Invitrogen) to photograph the cell density. After that, the cells were detached and counted by Countstar® BioTech automated cell counter.

Cell scratch migration assay was assessed using 24‐well plate. Briefly, cells were seeded at a density of 1 × 10^5^ cells per well and scraped using a sterile p200 pipette tip. Floating cells were cleaned with PBS washing. Serum‐free DMEM was applied to each well for 12 h. Analysis of wound repair was measured in three fields of view from the ratio of the closure area to the initial wound using the ImageJ. Live cell tracking of wound healing dynamics was performed for 3 days incubation on the IN Cell Analyzer 2500HS (GE) with a 10x/0.45 Plan Apo objective (Nikon) under the controlled atmosphere (5% CO_2_ and 20% O_2_) and constant temperature at 37°C. Phase‐contrast time‐lapse videos were visually represented.

For transwell migration assay, 1 × 10^4^ cells were suspended into the upper chamber followed by the addition of serum‐free DMEM in the lower chamber. After 12 h, we removed the cells remained on the upper surface membrane by cotton swab. Then a 0.5% crystal violet solution was used to stain the migrated cells on the lower side of the membrane for 1 h. The invasive cells attached on the lower chamber were stained with Hoechst 33342 at 37°C. Migrated and invasive cells were photographed and counted under EOVS M7000 microscope (Invitrogen).

Live/dead cells subjected to doxorubicin (Dox) treatment for 24 h (200 nM; Cat. No. D1515, Sigma‐Aldrich) were evaluated by JC‐1 Staining (Cat. No. C2005, Beyotime) and Mitochondrial Membrane Potential/Annexin V Apoptosis Kit (Cat. No. V35116, Invitrogen), respectively, following the manufacturer's instructions. For JC‐1, red staining captured by fluorescence microscope (DMI8, Leica) indicated more aggregate with JC‐1, which was indicative of hyperpolarized electrochemical membrane potential. By comparing the red/green ratio of excitation/emission 525/585 nm, relative levels of mitochondrial membrane potential were evaluated from 10^4^ cells in triplicate. For Annexin V assay, the stained cells were analyzed by flow cytometry (NovoCyte; ACEA) to calculate the apoptosis percentages. Fluorescence was measured at excitation/emission 579/599 nm with MitoTracker Red CMXRo and excitation/emission 499/521 nm with Alexa® Fluor 488 annexin V

Senescence associated β‐galactosidase (SA‐β‐Gal) activity subjected to Dox treatment was measured as described.^62^ Briefly, cells fixed with 4% paraformaldehyde were incubated in SA‐β‐Gal staining solution (Cat. No. C0602, Beyotime) for 12 h at 37°C. Cellular senescence was showed as the proportion of blue SA‐β‐Gal positive cells relative to the total cell number.

For colony formation after Dox treatment, ~ 100 of cells were seeded in triplicate in each well of six‐well plates and left for 14 days in the incubator to allow colony formation. Colonies were stained with 10% methylene blue in 70% ethanol. The surviving fraction was indicated as the ratio of blue staining area to the total well area.

For osteogenic differentiation, the cells were expanded in commercial osteogenic medium (Cat. No. SCM121, Sigma‐Aldrich). On Day 3 of culture, the ALP activity of cells was measured using a detection kit (Cat. No. P0321, Beyotime) by calculating the positive rates of ALP staining in the total cell numbers. On Day 7, the calcium deposits were discovered using the staining of Alizarin Red S (Cat. No. A5533, Sigma‐Aldrich). For adipogenic induction, on day 14 of culture in adipogenic medium (Cat. No. SCM122, Sigma‐Aldrich), cells were fixed in 4% paraformaldehyde and washed by 60% isopropanol and stained them with Oil red O solution (Cat. No. O0625, Sigma‐Aldrich) for 10 min at room temperature, followed by repeated washing. Imaged cells were quantified using ImageJ.^63^ Chondrogenesis was evaluated using U‐bottom suspension culture system described previously.^64^ Briefly, chondrocyte pellets were formed during 21 days culture in chondrogenic differentiation medium (Cat. No. SCM123, Sigma‐Aldrich), followed by staining with Alcian Blue (Cat. No. A5268, Sigma‐Aldrich). The optical density of the photograph was analyzed using ImageJ.

### 4.11 The efficacy of mitochondria transferred ADSCs in wound healing

To avoid the immune response to human cells transplantation in animal model, mito‐transferred rat‐ADSCs were engineered for the following in vivo study using the optimized method mentioned above. Briefly, adipose tissue was obtained from the right inguinal region of Sprague Dawley (SD) Rat A and B (male, 3 weeks, ~200 g), respectively. Donor Rat A‐ADSCs were expanded to isolate the mitochondria using the same method as described above. Mito‐transferred rat‐ADSCs were constructed by co‐culture with exogenous mitochondria at 18.5 μg/ml per 10^4^ of serum‐starved Rat B‐ADSCs for 6 h.

Wound healing experiments by full‐thickness excision were performed based on a reported protocol.^65^ SD rats (male, 3 weeks, ~200 g, *n* = 12) were used. The Ethics Committee of Zhejiang University (Ethics Code. ZJU20200166) legally approved all animal operations and experimental procedures. Anesthesia of rat was induced with 7% chloral hydrate. After shaving, depilatory cream was applied on the dorsal surface of the skin to remove hair completely. Two full‐thickness dorsal excisional skin wounds were made for each rat using a sterile 10 mm‐diameter biopsy punch. The rats were divided into three groups: a saline group, a nontreated ADSCs group, and a mito‐transferred ADSCs group. Total 6 × 10^6^ cells suspended in 1 ml PBS were locally injected into the fat layer of wound (*n* = 8 for each group). Rats were returned to cage after the recovery from anesthesia and daily monitored to evaluate health.

The distribution of mito‐transferred ADSCs after 6 h post‐transplantation was examined using in vivo imaging (IVIS Spectrum) by detecting MitoTracker Red CMXRo labelled exogenous Rat A‐derived mitochondria. The existence of exogenous Rat A‐derived mitochondria was also checked its fluorescence using tissue section. Briefly, the fixed adipose tissue was embedded in optimal cutting temperature (OCT) compound and frozen at −70°C. The frozen samples were sliced into 20 μm‐thick sections using Leica CM 1950 cryostat and processed them for fluorescence scanning using Eclipse Ni‐U microscope (Nikon). Later, adipocytes were further permeabilized with 0.5% Triton‐X100 (Cat. No. T8787, Sigma‐Aldrich) in PBS for 30 min and blocked with 5% bovine serum albumin in PBS for 1 h at room temperature. Cell nuclei were stained with DAPI (Cat. No. D9542, Sigma‐Aldrich) in PBS for 30 min at room temperature, and filamentous actin was labeled by using phalloidin‐iFluor 488 (Cat. No. ab176753, Abcam) for 1 h. Immunofluorescence images were acquired with Zeiss LSM 880 confocal microscope with 20× objective.

To correct the distance between the animals and the camera, a ruler reference was placed when taking the images. After determining the area of the reference circle by ImageJ, the wound area was quantified and described as a percentage of the original area. The wound area was photographed on the day of surgery, Day 3, Day 7, and Day 14 until fully healed. The surrounding tissues were also collected on Days 7 and 14 for histological analysis. Briefly, the skins were fixed in 4% paraformaldehyde followed by dehydrating with a graded ethanol series. After imbedding in Paraffin, 7 μm‐thick sections were sliced, deparaffinized and finally stained with masson trichrome stain and H&E stain. The stained tissue sections were imaged with Pannoramic MIDI II (3DHISTECH). The degree of re‐epithelialization and collagen synthesis in different groups was estimated by a blinded histopathology expert using a semi‐quantitative scoring system based on histological sections, which is scored with a 5‐point scale (0, thickness of cut edges and absent granulation tissue; 1, migration of cells [<50%] and minimal granulation tissue; 2, migration of cells [≥50%] and mild granulation tissue; 3, bridging the excision and moderate granulation tissue; and 4, keratinization and marked granulation tissue).

### 4.12 Statistical analysis

The quantitative data were expressed as means ± SD. Two‐tailed Student's *t* tests were conducted to analyze the statistical differences between two groups, while the statistical differences among multiple groups were analyzed by analysis of variance (ANOVA) using GraphPad Prism 7.04 (San Diego, USA). The *p* values are shown in the figures as *p < 0.05, **p < 0.01, and ***p < 0.001.

## AUTHOR CONTRIBUTIONS

**Hongwei Ouyang:** Conceptualization (lead); funding acquisition (lead); supervision (lead). **Xudong Yao:** Funding acquisition (lead); investigation (lead); methodology (equal); validation (lead); writing – original draft (equal); writing – review and editing (equal). **Yuanzhu Ma:** Investigation (equal); methodology (equal); validation (equal); writing – original draft (equal); writing – review and editing (equal). **Wenyan Zhou:** Investigation (equal); methodology (equal); writing – original draft (equal); writing – review and editing (equal). **Youguo Liao:** Investigation (supporting); methodology (equal). **Zongsheng Jiang:** Investigation (supporting); methodology (supporting). **Junxin Lin:** Investigation (supporting); methodology (equal); software (equal); validation (supporting). **Qiulin He:** Resources (supporting); software (equal). **Hongwei Wu:** Investigation (supporting); methodology (supporting). **Wei Wei:** Investigation (supporting); methodology (supporting). **Xiaozhao Wang:** Investigation (supporting); methodology (supporting). **Mikael Björklund:** Writing – review and editing (supporting).

## CONFLICT OF INTERESTS

The authors declare that they have no known competing financial interests or personal relationships that could have appeared to influence the work reported in this paper.

### PEER REVIEW

The peer review history for this article is available at https://publons.com/publon/10.1002/btm2.10250.

## Supporting information


**Video S1.** Absence of exogenous mitochondria

**Video S2.** Low mitochondrial dose delivered

**Video S3.** Moderate mitochondrial dose delivered

**Video S4.** High mitochondrial dose delivered

**Video S5.** Superior migratory ability

**Figure S1** Identification of donor Y40‐ADSCs and recipient Y74‐ADSCs. (A) and (B) Flow cytometric analysis showed that express specific markers for ADSCs such as CD73, CD90 and CD105; absence of CD34 and CD45. Green open histogram represented the control, and red open histogram represented the antibodies (n=3 per group).
**Figure S2** Comparison of bioenergetic status between Y40‐ and Y74‐ADSCs. (A) Mitochondrial distribution and representative TEM image of intracellular mitochondria in 740‐ADSCs. Scale bar, 10 μm and 0.2 μm, respectively. (B) The total amount of ATP produced from Y40‐ADSCs and Y74‐ADSCs with a population of 1 x 10^6^ cells. (C) Relative quantification of the copy numbers of ND1/SLCO2B1 and ND5/SERPINA1 by RT‐PCR in Y40‐ADSCs and Y74‐ADSCs. ND1 and ND5 pairs for the detection of mitochondrial DNA (mtDNA), and SLCO2B1 and SERPINA1 pairs for the detection of nuclear DNA (nDNA). Significantly different (one‐way ANOVA): ns, not significant.
**Figure S3** Validation of mitochondrial isolation. (A) Flow cytometric analysis confirmed the complete cell disruption after chemical and mechanical lysis. (B) Quantification of mitochondrial DNA (mtDNA) isolated from different cell number. (C) Quantification of mitochondrial protein (mito‐protein) isolated from different cell number. (D) Mitochondrial ATP was kept constant at 6.2 μM in per unit of mito‐protein. Significantly different (one‐way ANOVA): ns, not significant, **P < 0.01, and ***P < 0.001.
**Figure S4** Evolution of oxidative stress by levels of 8‐OHdG in Y74‐ADSCs after mitochondrial uptake (n=3). Significantly different (one‐way ANOVA): ns, not significant.
**Figure S5** The improved cell‐migration of Y74‐ADSCs after mitochondrial uptake. (A) and (B) Representative images and quantification of control Y74‐ADSCs and mito transferred Y74‐ADSCs cell‐migration (n=3). Scale bars, 100 μm.
**Figure S6** The superior cell‐invasion of Y74‐ADSCs after mitochondrial uptake. (A) and (B) Representative images and quantification of control Y74‐ADSCs and mito transferred Y74‐ADSCs cell‐invasion (n=3). Scale bars, 50 μm.
**Figure S7** The promoted survival rate of Y74‐ADSCs against Dox treatment after mitochondrial transfer. (A) CCCP as negative control induced complete cell death. Dox decreased mitochondrial transmembrane electric potential subjected to fluorescence microscopy (Magenta, JC‐1 aggregates; green, JC‐1 monomers). Scale bars, 20 μm. (B) The relative levels of mitochondrial membrane potential were calculated by the ratio between the fluorescence intensity obtained at red fluorescence of energized mitochondrion and green fluorescence of de‐energized mitochondrion. (C) Flow cytometric analysis on the apoptosis levels of non‐treated Y74‐ADSCs and mito transferred Y74‐ADSCs after Dox treatment. The incubation condition is: 200 nM Dox for 12 hours. Significantly different (one‐way ANOVA): *P < 0.05.
**Figure S8** The percentages of wound area healed relative to the original wound by different treatments at days 3, 7, and 14 (n=8). Significantly different (one‐way ANOVA): *P < 0.05, and ***P < 0.001.

**Table S1** List of 58 highly expressed proteins in mito‐transferred Y74‐ADSCs CM as compared to control Y74‐ADSCs CM (log_2_FC > 2).

## Data Availability

All data needed to evaluate the conclusions in the paper are present in the paper and/or the Supplementary Materials. Additional data related to this paper may be requested from the authors.

## References

[1] Sarkar D , Spencer JA , Phillips JA , et al. Engineered cell homing. Blood. 2011;118(25):e184‐e191. 22034631 10.1182/blood-2010-10-311464 PMC3242725

[2] Fu Y , Ni J , Chen J , et al. Dual‐functionalized MSCs that express CX3CR1 and IL‐25 exhibit enhanced therapeutic effects on inflammatory bowel disease. Mol Ther. 2020;28(4):1214‐1228. 32087149 10.1016/j.ymthe.2020.01.020 PMC7132625

[3] Golinelli G , Grisendi G , Prapa M , et al. Targeting GD2‐positive glioblastoma by chimeric antigen receptor empowered mesenchymal progenitors. Cancer Gene Ther. 2020;27(7):558‐570. 30464207 10.1038/s41417-018-0062-x PMC7445885

[4] Srifa W , Kosaric N , Amorin A , et al. Cas9‐AAV6‐engineered human mesenchymal stromal cells improved cutaneous wound healing in diabetic mice. Nat Commun. 2020;11(1):2470. 32424320 10.1038/s41467-020-16065-3 PMC7235221

[5] Pezzi A , Amorin B , Laureano Á , et al. Effects of hypoxia in long‐term in vitro expansion of human bone marrow derived mesenchymal stem cells. J Cell Biochem. 2017;118(10):3072‐3079. 28240374 10.1002/jcb.25953

[6] Bernardo ME , Fibbe WE . Mesenchymal stromal cells: sensors and switchers of inflammation. Cell Stem Cell. 2013;13(4):392‐402. 24094322 10.1016/j.stem.2013.09.006

[7] Levy O , Mortensen LJ , Boquet G , et al. A small‐molecule screen for enhanced homing of systemically infused cells. Cell Rep. 2015;10(8):1261‐1268. 25732817 10.1016/j.celrep.2015.01.057 PMC4361231

[8] Levy O , Zhao W , Mortensen LJ , et al. mRNA‐engineered mesenchymal stem cells for targeted delivery of interleukin‐10 to sites of inflammation. Blood. 2013;122(14):e23‐e32. 23980067 10.1182/blood-2013-04-495119 PMC3790516

[9] Ankrum JA , Miranda OR , Ng KS , Sarkar D , Xu C , Karp JM . Engineering cells with intracellular agent‐loaded microparticles to control cell phenotype. Nat Protoc. 2014;9(2):233‐245. 24407352 10.1038/nprot.2014.002 PMC4320648

[10] Yanai A , Häfeli UO , Metcalfe AL , et al. Focused magnetic stem cell targeting to the retina using superparamagnetic iron oxide nanoparticles. Cell Transplant. 2012;21(6):1137‐1148. 22405427 10.3727/096368911X627435

[11] Führmann T , Tam RY , Ballarin B , et al. Injectable hydrogel promotes early survival of induced pluripotent stem cell‐derived oligodendrocytes and attenuates longterm teratoma formation in a spinal cord injury model. Biomaterials. 2016;83:23‐36. 26773663 10.1016/j.biomaterials.2015.12.032

[12] Wu Z , Chen G , Zhang J , et al. Treatment of myocardial infarction with gene‐modified mesenchymal stem cells in a small molecular hydrogel. Sci Rep. 2017;7(1):15826. 29158523 10.1038/s41598-017-15870-z PMC5696474

[13] Ringden O , Baygan A , Remberger M , et al. Placenta‐derived decidua stromal cells for treatment of severe acute graft‐versus‐host disease. Stem Cells Transl Med. 2018;7(4):325‐331. 29533533 10.1002/sctm.17-0167 PMC5866941

[14] Baygan A , Aronsson‐Kurttila W , Moretti G , et al. Safety and side effects of using placenta‐derived decidual stromal cells for graft‐versus‐host disease and hemorrhagic cystitis. Front Immunol. 2017;8:795. 28744284 10.3389/fimmu.2017.00795 PMC5504152

[15] Park B‐W , Jung S‐H , Das S , et al. In vivo priming of human mesenchymal stem cells with hepatocyte growth factor–engineered mesenchymal stem cells promotes therapeutic potential for cardiac repair. Sci Adv. 2020;6(13):eaay6994. 32284967 10.1126/sciadv.aay6994 PMC7141892

[16] Spees JL , Olson SD , Whitney MJ , Prockop DJ . Mitochondrial transfer between cells can rescue aerobic respiration. Proc Natl Acad Sci U S A. 2006;103(5):1283‐1288. 16432190 10.1073/pnas.0510511103 PMC1345715

[17] Zhang Y , Yu Z , Jiang D , et al. iPSC‐MSCs with high intrinsic MIRO1 and sensitivity to TNF‐α yield efficacious mitochondrial transfer to rescue anthracycline‐induced cardiomyopathy. Stem Cell Rep. 2016;7(4):749‐763. 10.1016/j.stemcr.2016.08.009 PMC5063626 27641650

[18] Babenko VA , Silachev DN , Zorova LD , et al. Improving the post‐stroke therapeutic potency of mesenchymal multipotent stromal cells by cocultivation with cortical neurons: the role of crosstalk between cells. Stem Cells Transl Med. 2015;4(9):1011‐1020. 26160961 10.5966/sctm.2015-0010 PMC4542870

[19] Moschoi R , Imbert V , Nebout M , et al. Protective mitochondrial transfer from bone marrow stromal cells to acute myeloid leukemic cells during chemotherapy. Blood. 2016;128(2):253‐264. 27257182 10.1182/blood-2015-07-655860

[20] Jackson MV , Morrison TJ , Doherty DF , et al. Mitochondrial transfer via tunneling nanotubes is an important mechanism by which mesenchymal stem cells enhance macrophage phagocytosis in the in vitro and in vivo models of ARDS. Stem Cells. 2016;34(8):2210‐2223. 27059413 10.1002/stem.2372 PMC4982045

[21] Jiang D , Gao F , Zhang Y , et al. Mitochondrial transfer of mesenchymal stem cells effectively protects corneal epithelial cells from mitochondrial damage. Cell Death Dis. 2016;7(11):e2467. 27831562 10.1038/cddis.2016.358 PMC5260876

[22] Li X , Zhang Y , Yeung SC , et al. Mitochondrial transfer of induced pluripotent stem cell‐derived mesenchymal stem cells to airway epithelial cells attenuates cigarette smoke‐induced damage. Am J Respir Cell Mol Biol. 2014;51(3):455‐465. 24738760 10.1165/rcmb.2013-0529OC

[23] Paliwal S , Chaudhuri R , Agrawal A , Mohanty S . Regenerative abilities of mesenchymal stem cells through mitochondrial transfer. J Biomed Sci. 2018;25(1):31. 29602309 10.1186/s12929-018-0429-1 PMC5877369

[24] Guariento A , Blitzer D , Doulamis I , et al. Preischemic autologous mitochondrial transplantation by intracoronary injection for myocardial protection. J Thorac Cardiovasc Surg. 2020;160(2):e15‐e29. 31564546 10.1016/j.jtcvs.2019.06.111

[25] McCully JD , Cowan DB , Emani SM , Del Nido PJ . Mitochondrial transplantation: from animal models to clinical use in humans. Mitochondrion. 2017;34:127‐134. 28342934 10.1016/j.mito.2017.03.004

[26] Emani SM , Piekarski BL , Harrild D , Del Nido PJ , McCully JD . Autologous mitochondrial transplantation for dysfunction after ischemia‐reperfusion injury. J Thorac Cardiovasc Surg. 2017;154(1):286‐289. 28283239 10.1016/j.jtcvs.2017.02.018

[27] Loebel C , Burdick JA . Engineering stem and stromal cell therapies for musculoskeletal tissue repair. Cell Stem Cell. 2018;22(3):325‐339. 29429944 10.1016/j.stem.2018.01.014 PMC5834383

[28] Levy O , Kuai R , Siren EMJ , et al. Shattering barriers toward clinically meaningful MSC therapies. Sci Adv. 2020;6(30):eaba6884. 32832666 10.1126/sciadv.aba6884 PMC7439491

[29] Almeida‐Porada G , Atala AJ , Porada CD . Therapeutic mesenchymal stromal cells for immunotherapy and for gene and drug delivery. Mol Ther. 2020;16:204‐224. 10.1016/j.omtm.2020.01.005 PMC7012781 32071924

[30] Zhang T , Xu Q , Huang T , Ling D , Gao J . New insights into biocompatible iron oxide nanoparticles: a potential booster of gene delivery to stem cells. Small. 2020;16(37):2001588. 10.1002/smll.202001588 32725792

[31] Nasiri N , Hosseini S , Alini M , Khademhosseini A , Baghaban Eslaminejad M . Targeted cell delivery for articular cartilage regeneration and osteoarthritis treatment. Drug Discov Today. 2019;24(11):2212‐2224. 31398399 10.1016/j.drudis.2019.07.010

[32] Kim MJ , Hwang JW , Yun CK , Lee Y , Choi YS . Delivery of exogenous mitochondria via centrifugation enhances cellular metabolic function. Sci Rep. 2018;8(1):3330. 29463809 10.1038/s41598-018-21539-y PMC5820364

[33] Funes JM , Quintero M , Henderson S , et al. Transformation of human mesenchymal stem cells increases their dependency on oxidative phosphorylation for energy production. Proc Natl Acad USA. 2007;104(15):6223‐6228. 10.1073/pnas.0700690104 PMC1851087 17384149

[34] Warburg O . On the origin of cancer cells. Science. 1956;123(3191):309‐314. 13298683 10.1126/science.123.3191.309

[35] Vander Heiden MG , Cantley LC , Thompson CB . Understanding the Warburg effect: the metabolic requirements of cell proliferation. Science. 2009;324(5930):1029‐1033. 19460998 10.1126/science.1160809 PMC2849637

[36] Vaux EC , Metzen E , Yeates KM , Ratcliffe PJ . Regulation of hypoxia‐inducible factor is preserved in the absence of a functioning mitochondrial respiratory chain. Blood. 2001;98(2):296‐302. 11435296 10.1182/blood.v98.2.296

[37] Wang S , Shi X , Wei S , et al. Krüppel‐like factor 4 (KLF4) induces mitochondrial fusion and increases spare respiratory capacity of human glioblastoma cells. J Biol Chem. 2018;293(17):6544‐6555. 29507094 10.1074/jbc.RA117.001323 PMC5925822

[38] Hill BG , Benavides GA , Lancaster JR Jr , et al. Integration of cellular bioenergetics with mitochondrial quality control and autophagy. Biol Chem. 2012;393(12):1485‐1512. 23092819 10.1515/hsz-2012-0198 PMC3594552

[39] Banh RS , Iorio C , Marcotte R , et al. PTP1B controls non‐mitochondrial oxygen consumption by regulating RNF213 to promote tumour survival during hypoxia. Nat Cell Biol. 2016;18(7):803‐813. 27323329 10.1038/ncb3376 PMC4936519

[40] Chi Y , Carter JH , Swanger J , Mazin AV , Moritz RL , Clurman BE . A novel landscape of nuclear human CDK2 substrates revealed by in situ phosphorylation. Sci Adv. 2020;6(16):eaaz9899. 32494624 10.1126/sciadv.aaz9899 PMC7164936

[41] Rhee J‐W , Wu JC . Cardiac cell cycle activation as a strategy to improve iPSC‐derived cardiomyocyte therapy. Circul Res. 2018;122(1):14‐16. 10.1161/CIRCRESAHA.117.312287 PMC5773069 29301838

[42] McDevitt TC , Laflamme MA , Murry CE . Proliferation of cardiomyocytes derived from human embryonic stem cells is mediated via the IGF/PI 3‐kinase/Akt signaling pathway. J Mol Cell Cardiol. 2005;39(6):865‐873. 16242146 10.1016/j.yjmcc.2005.09.007 PMC3505759

[43] Wang XQ , Lo CM , Chen L , Ngan ES , Xu A , Poon RY . CDK1‐PDK1‐PI3K/Akt signaling pathway regulates embryonic and induced pluripotency. Cell Death Differ. 2017;24(1):38‐48. 27636107 10.1038/cdd.2016.84 PMC5260505

[44] Liu L , Michowski W , Kolodziejczyk A , Sicinski P . The cell cycle in stem cell proliferation, pluripotency and differentiation. Nat Cell Biol. 2019;21(9):1060‐1067. 31481793 10.1038/s41556-019-0384-4 PMC7065707

[45] Díez‐Juan A , Andrés V . Coordinate control of proliferation and migration by the p27Kip1/cyclin‐dependent kinase/retinoblastoma pathway in vascular smooth muscle cells and fibroblasts. Circ Res. 2003;92(4):402‐410. 12600894 10.1161/01.RES.0000059306.71961.ED

[46] Zhu W , Zhao M , Mattapally S , Chen S , Zhang J . CCND2 overexpression enhances the regenerative potency of human induced pluripotent stem cellderived cardiomyocytes: remuscularization of injured ventricle. Circ Res. 2018;122(1):88‐96. 29018036 10.1161/CIRCRESAHA.117.311504 PMC5756126

[47] Ahmed AA , Mills AD , Ibrahim AE , et al. The extracellular matrix protein TGFBI induces microtubule stabilization and sensitizes ovarian cancers to paclitaxel. Cancer Cell. 2007;12(6):514‐527. 18068629 10.1016/j.ccr.2007.11.014 PMC2148463

[48] Davidsen ML , Würtz S , Rømer MU , et al. TIMP‐1 gene deficiency increases tumour cell sensitivity to chemotherapy‐induced apoptosis. Brit J Cancer. 2006;95(8):1114‐1120. 17047657 10.1038/sj.bjc.6603378 PMC2360707

[49] Li CX , Talele NP , Boo S , et al. MicroRNA‐21 preserves the fibrotic mechanical memory of mesenchymal stem cells. Nat Mater. 2017;16(3):379‐389. 27798620 10.1038/nmat4780

[50] McLeod CM , Mauck RL . On the origin and impact of mesenchymal stem cell heterogeneity: new insights and emerging tools for single cell analysis. Eur Cell Mater. 2017;34:217‐231. 29076514 10.22203/eCM.v034a14 PMC7735381

[51] Bertero E , O'Rourke B , Maack C . Mitochondria do not survive calcium overload during transplantation. Circ Res. 2020;126(6):784‐786. 32078444 10.1161/CIRCRESAHA.119.316291 PMC7781225

[52] Bernardi P , Rasola A , Forte M , Lippe G . The mitochondrial permeability transition pore: channel formation by F‐ATP synthase, integration in signal transduction, and role in pathophysiology. Physiol Rev. 2015;95(4):1111‐1155. 26269524 10.1152/physrev.00001.2015 PMC4600949

[53] Lansdown AB . Calcium: a potential central regulator in wound healing in the skin. Wound Repair Regen. 2002;10(5):271‐285. 12406163 10.1046/j.1524-475x.2002.10502.x

[54] Liu X , Xiang Q , Xu F , et al. Single‐cell RNA‐seq of cultured human adipose‐derived mesenchymal stem cells. Sci Data. 2019;6(1):190031. 30806636 10.1038/sdata.2019.31 PMC6390702

[55] Kamogashira T , Hayashi K , Fujimoto C , Iwasaki S , Yamasoba T . Functionally and morphologically damaged mitochondria observed in auditory cells under senescence‐inducing stress. NPJ Aging Mech Dis. 2017;3:2‐2. 28649420 10.1038/s41514-017-0002-2 PMC5445612

[56] Varkuti BH , Kepiro M , Liu Z , et al. Neuron‐based high‐content assay and screen for CNS active mitotherapeutics. Sci Adv. 2020;6(2):eaaw8702. 31934620 10.1126/sciadv.aaw8702 PMC6949038

[57] Ali Pour P , Kenney MC , Kheradvar A . Bioenergetics consequences of mitochondrial transplantation in cardiomyocytes. J Am Heart Assoc. 2020;9(7):e014501. 32200731 10.1161/JAHA.119.014501 PMC7428632

[58] Chambers KM , Mandavilli BS , Dolman NJ , Janes MS . General staining and segmentation procedures for high content imaging and analysis. Methods Mol Biol. 2018;1683:21‐31. 29082484 10.1007/978-1-4939-7357-6_2

[59] Efstathiou G , Antonakis AN , Pavlopoulos GA , et al. ProteoSign: an end‐user online differential proteomics statistical analysis platform. Nucl Acids Res. 2017;45(W1):W300‐W306. 28520987 10.1093/nar/gkx444 PMC5793730

[60] Love MI , Huber W , Anders S . Moderated estimation of fold change and dispersion for RNA‐seq data with DESeq2. Genome Biol. 2014;15(12):550. 25516281 10.1186/s13059-014-0550-8 PMC4302049

[61] Yu G , Wang L‐G , Han Y , He Q‐Y . clusterProfiler: an R package for comparing biological themes among gene clusters. OMICS. 2012;16(5):284‐287. 22455463 10.1089/omi.2011.0118 PMC3339379

[62] Shin E‐Y , Park J‐H , You S‐T , et al. Integrin‐mediated adhesions in regulation of cellular senescence. Sci Adv. 2020;6(19):eaay3909. 32494696 10.1126/sciadv.aay3909 PMC7202880

[63] Mehlem A , Hagberg CE , Muhl L , Eriksson U , Falkevall A . Imaging of neutral lipids by oil red O for analyzing the metabolic status in health and disease. Nat Protoc. 2013;8(6):1149‐1154. 23702831 10.1038/nprot.2013.055

[64] Wang Y , Zhang X , Huang H , et al. Osteocalcin expressing cells from tendon sheaths in mice contribute to tendon repair by activating Hedgehog signaling. Elife. 2017;6:1‐27. 10.7554/eLife.30474 PMC5731821 29244023

[65] Chen H , Cheng Y , Tian J , et al. Dissolved oxygen from microalgae‐gel patch promotes chronic wound healing in diabetes. Sci Adv. 2020;6(20):eaba4311. 32440554 10.1126/sciadv.aba4311 PMC7228761

