# Supplementary material for: In‐cytoplasm mitochondrial transplantation for mesenchymal stem cells engineering and tissue regeneration
Source: Bioeng Transl Med. 2021 Sep 28;7(1):e10250. doi: 10.1002/btm2.10250 (PMC8780934; doi:10.1002/btm2.10250)

**Supplementary figure legends**

Figure S1 Identification of donor Y40-ADSCs and recipient Y74-ADSCs. (A) and (B) Flow cytometric analysis showed that express specific markers for ADSCs such as CD73, CD90 and CD105; absence of CD34 and CD45. Green open histogram represented the control, and red open histogram represented the antibodies (n=3 per group).

Figure S2 Comparison of bioenergetic status between Y40- and Y74-ADSCs. (A) Mitochondrial distribution and representative TEM image of intracellular mitochondria in 740-ADSCs. Scale bar, 10 μm and 0.2 μm, respectively. (B) The total amount of ATP produced from Y40-ADSCs and Y74-ADSCs with a population of 1 x 10^6^ cells. (C) Relative quantification of the copy numbers of ND1/SLCO2B1 and ND5/SERPINA1 by RT-PCR in Y40-ADSCs and Y74-ADSCs. ND1 and ND5 pairs for the detection of mitochondrial DNA (mtDNA), and SLCO2B1 and SERPINA1 pairs for the detection of nuclear DNA (nDNA). Significantly different (one-way ANOVA): ns, not significant.

Figure S3 Validation of mitochondrial isolation. (A) Flow cytometric analysis confirmed the complete cell disruption after chemical and mechanical lysis. (B) Quantification of mitochondrial DNA (mtDNA) isolated from different cell number. (C) Quantification of mitochondrial protein (mito-protein) isolated from different cell number. (D) Mitochondrial ATP was kept constant at 6.2 μM in per unit of mito-protein. Significantly different (one-way ANOVA): ns, not significant, **P < 0.01, and ***P < 0.001.

Figure S4 Evolution of oxidative stress by levels of 8-OHdG in Y74-ADSCs after mitochondrial uptake (n=3). Significantly different (one-way ANOVA): ns, not significant.

Figure S5 The improved cell-migration of Y74-ADSCs after mitochondrial uptake. (A) and (B) Representative images and quantification of control Y74-ADSCs and mito transferred Y74-ADSCs cell-migration (n=3). Scale bars, 100 μm.

Figure S6 The superior cell-invasion of Y74-ADSCs after mitochondrial uptake. (A) and (B) Representative images and quantification of control Y74-ADSCs and mito transferred Y74-ADSCs cell-invasion (n=3). Scale bars, 50 μm.

Figure S7 The promoted survival rate of Y74-ADSCs against Dox treatment after mitochondrial transfer. (A) CCCP as negative control induced complete cell death. Dox decreased mitochondrial transmembrane electric potential subjected to fluorescence microscopy (Magenta, JC-1 aggregates; green, JC-1 monomers). Scale bars, 20 μm. (B) The relative levels of mitochondrial membrane potential were calculated by the ratio between the fluorescence intensity obtained at red fluorescence of energized mitochondrion and green fluorescence of de-energized mitochondrion. (C) Flow cytometric analysis on the apoptosis levels of non-treated Y74-ADSCs and mito transferred Y74-ADSCs after Dox treatment. The incubation condition is: 200 nM Dox for 12 hours. Significantly different (one-way ANOVA): *P < 0.05.

Figure S8 The percentages of wound area healed relative to the original wound by different treatments at days 3, 7, and 14 (n=8). Significantly different (one-way ANOVA): *P < 0.05, and ***P < 0.001.

**Figure S1
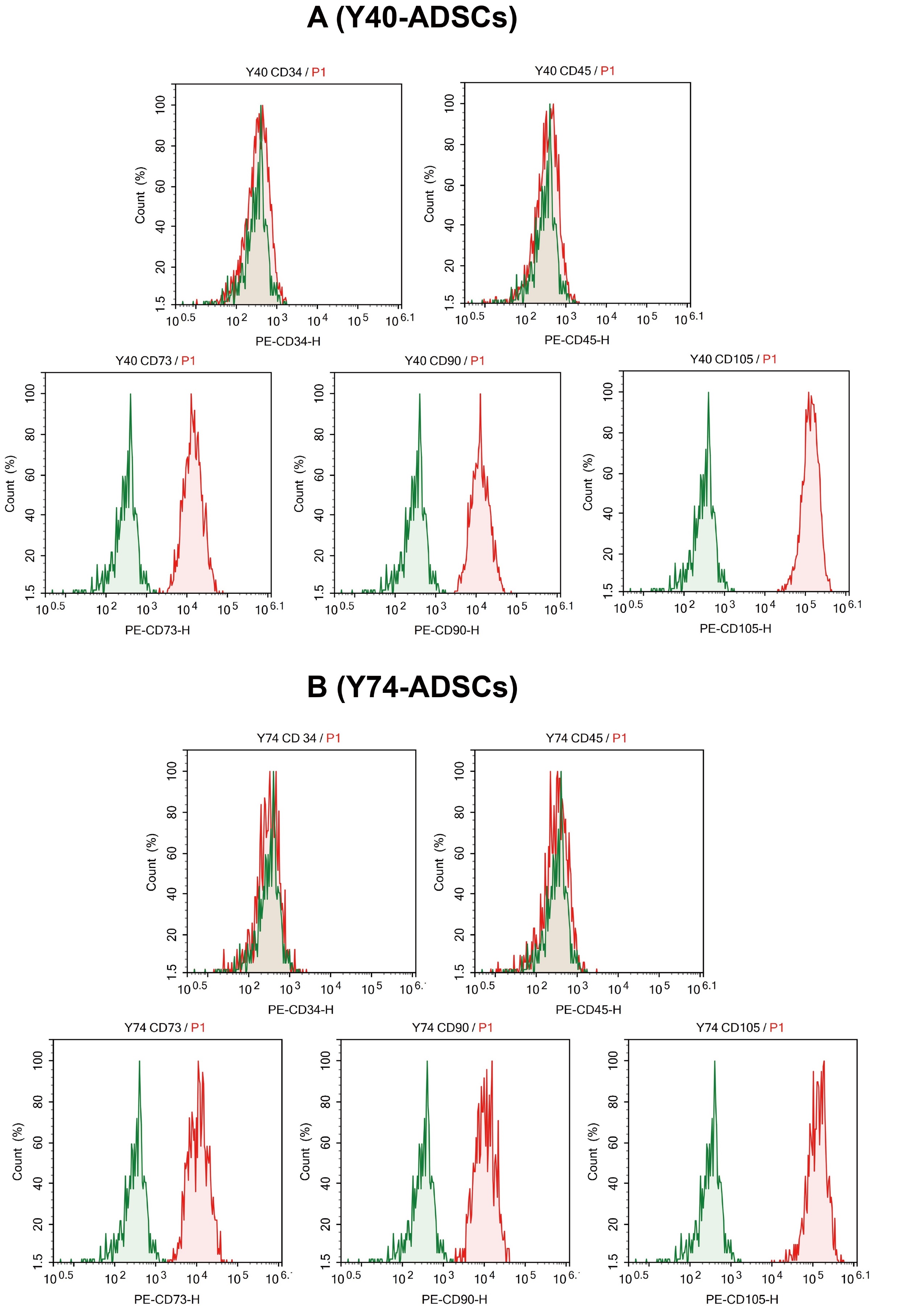
**

**Figure S2**

**
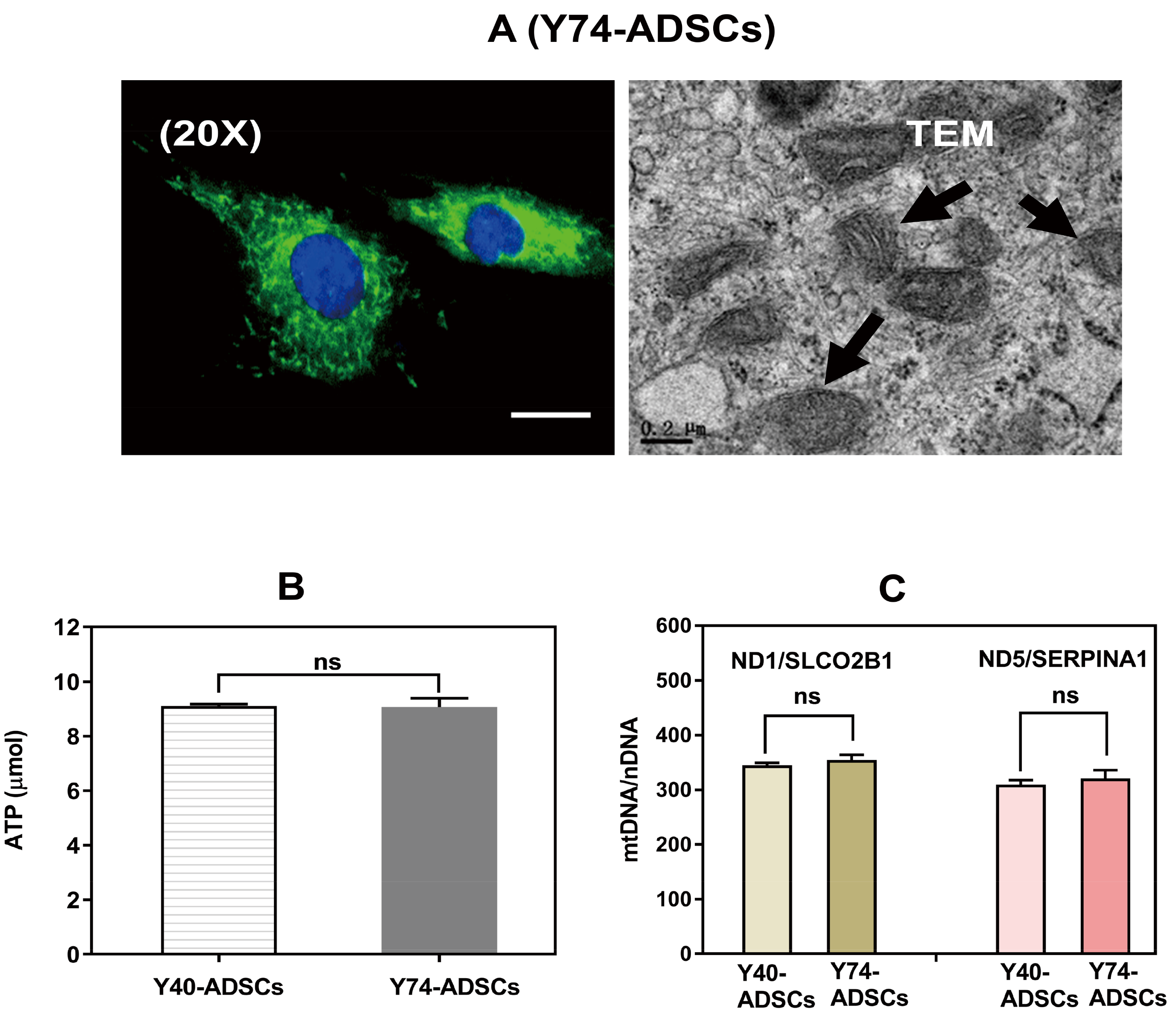
**

**Figure S3**


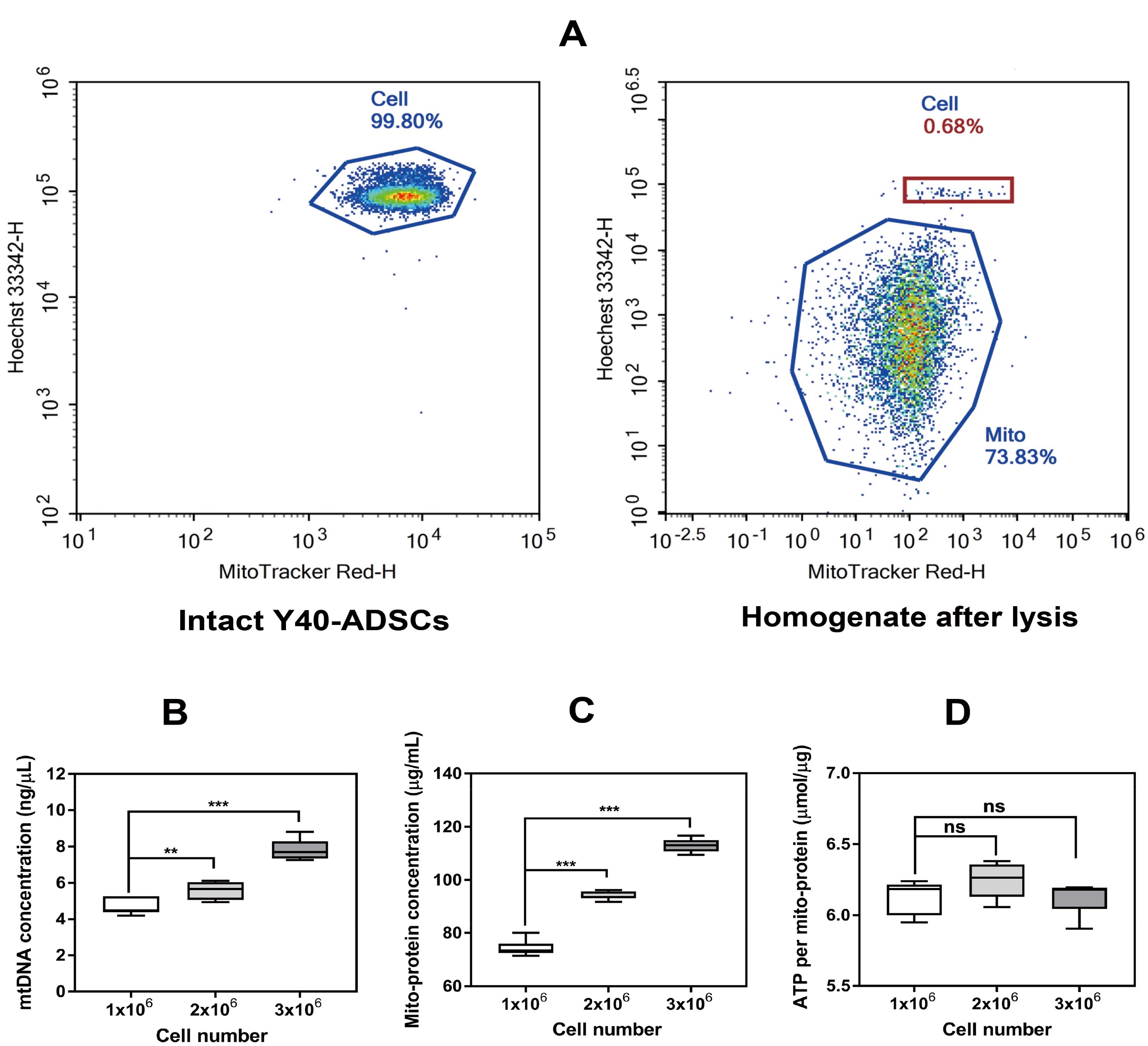


**Figure S4**


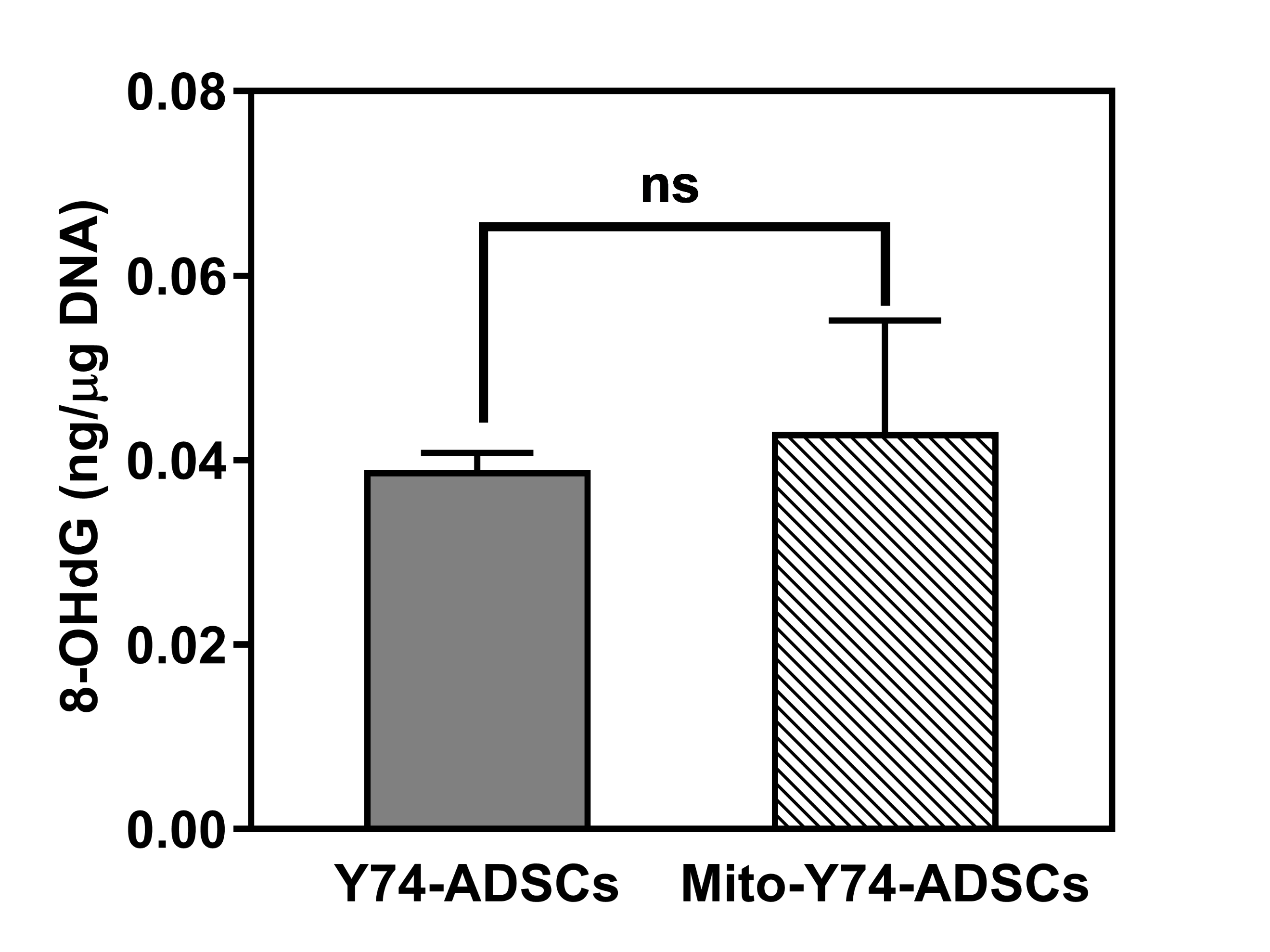


**Figure S5**
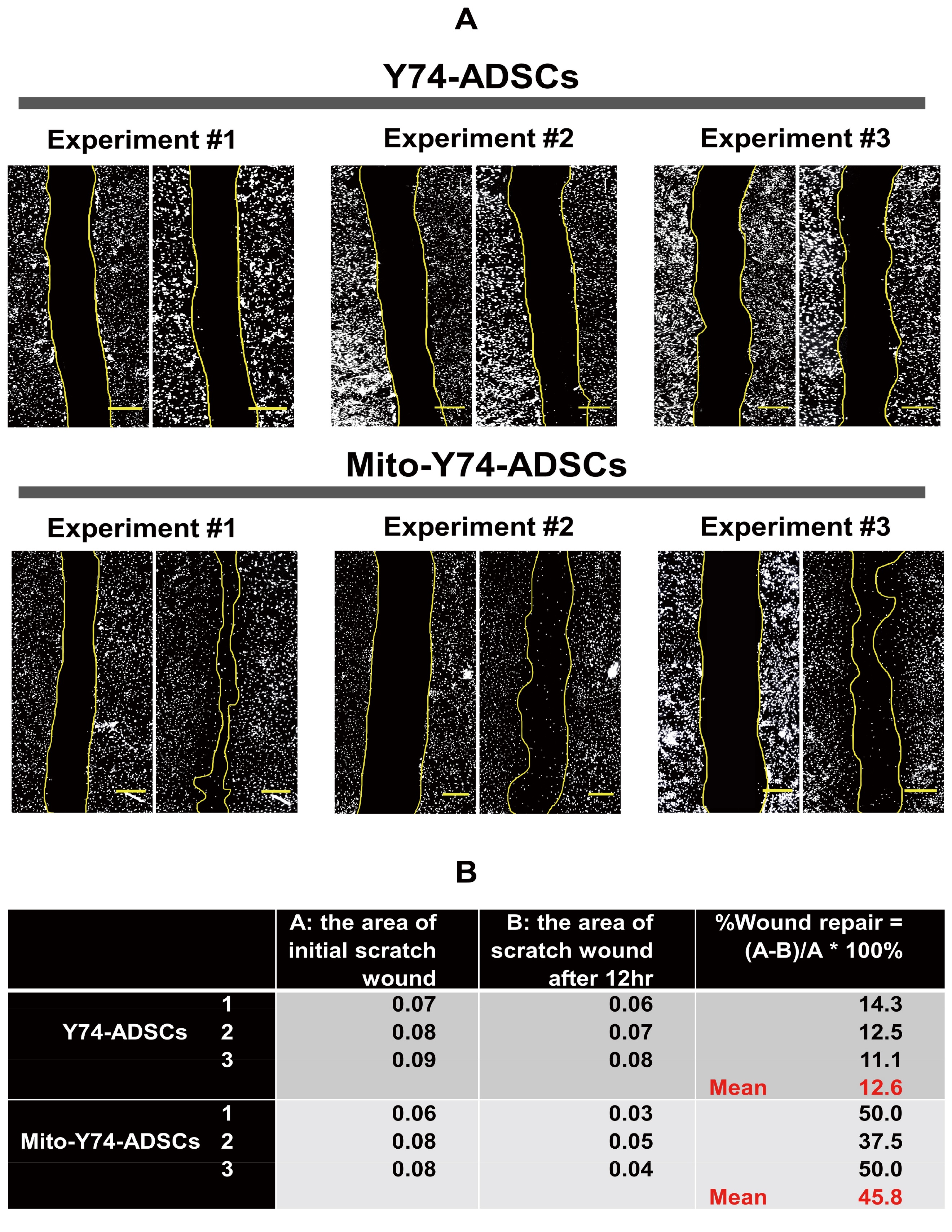


**Figure S6**

**
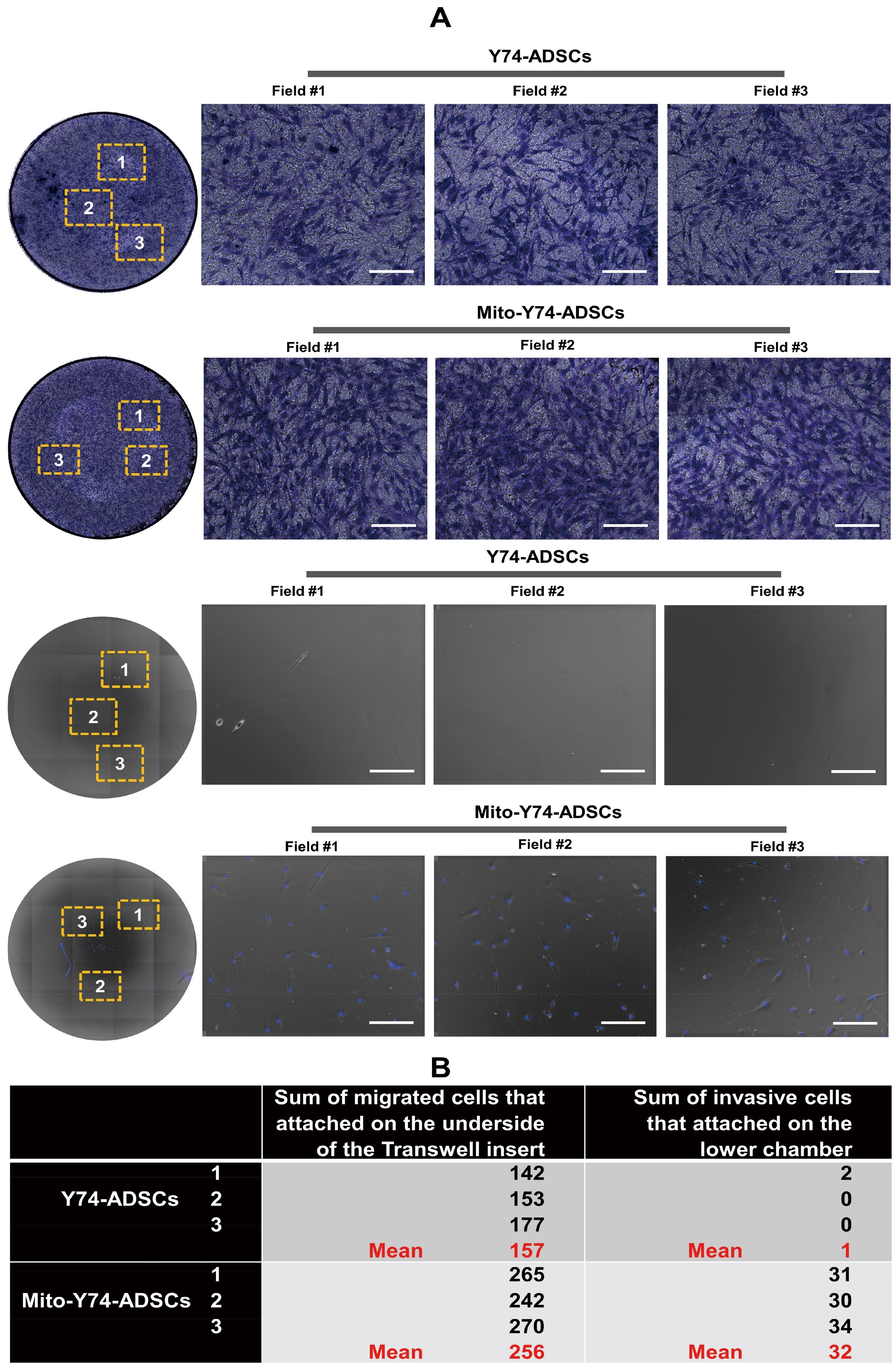
**

**Figure S7**


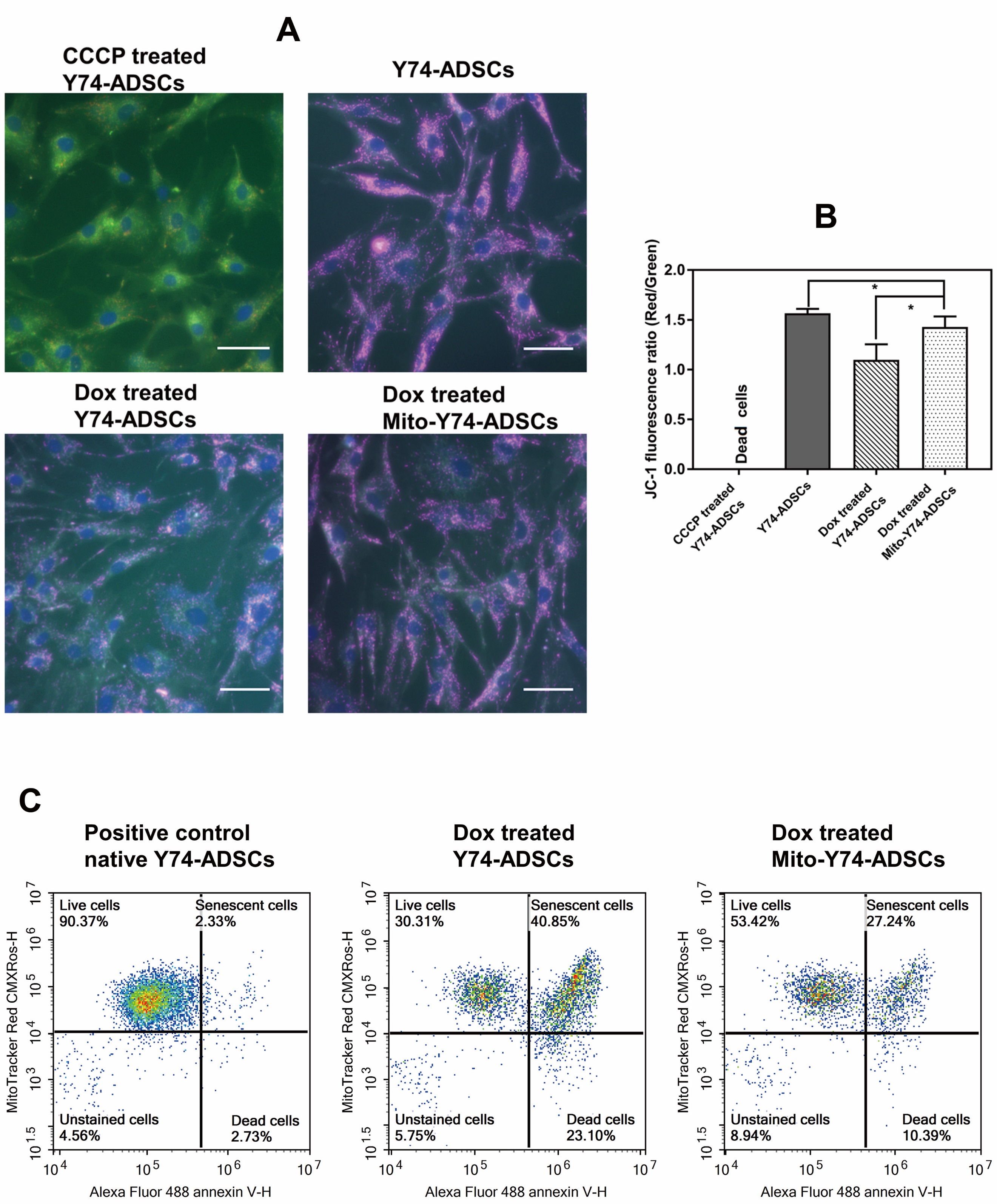


**Figure S8**


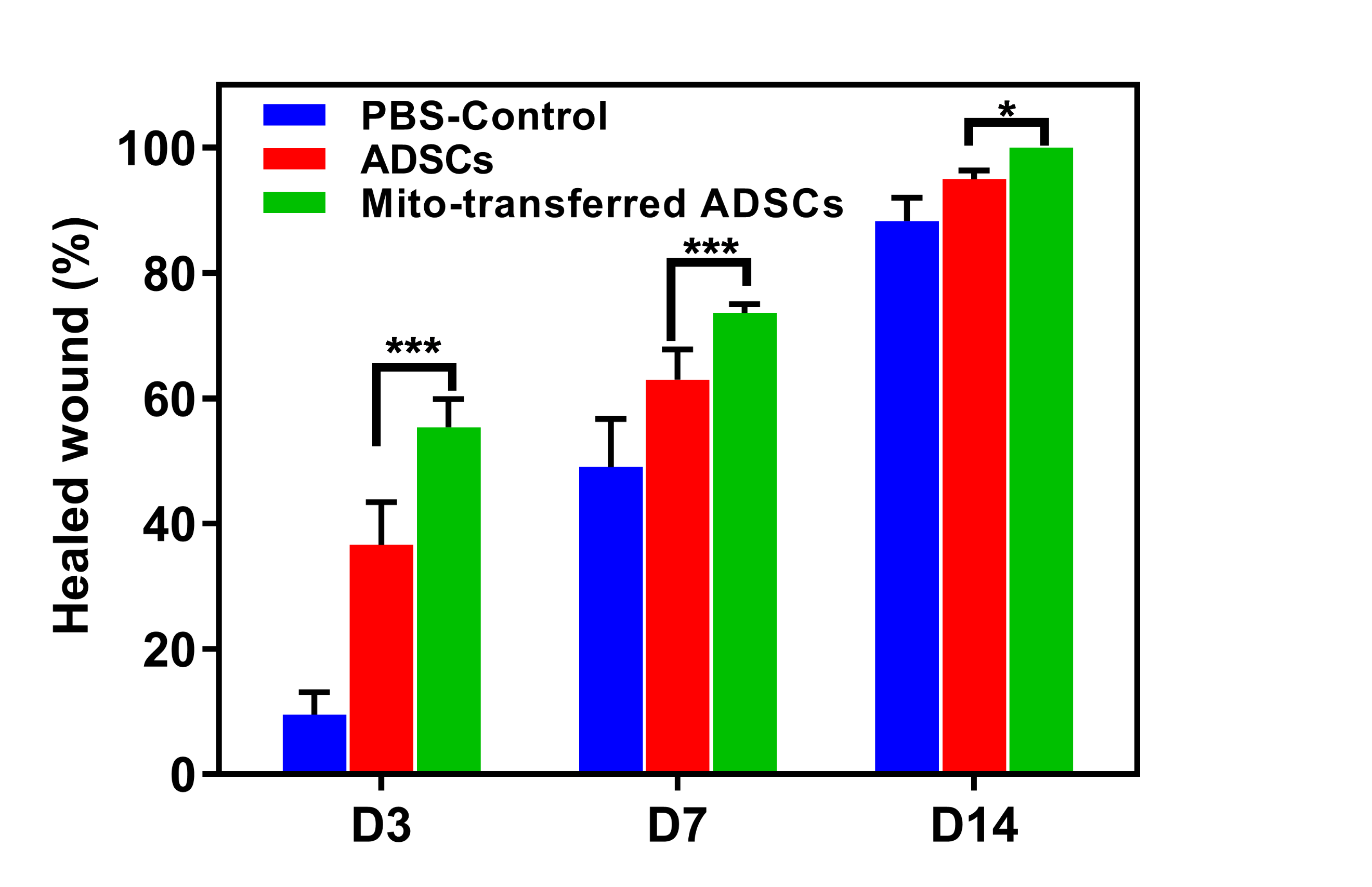

Supplement: Supplementary file 6 — Figure S1 Identification of donor Y40‐ADSCs and recipient Y74‐ADSCs. (A) and (B) Flow cytometric analysis showed that express specific markers for ADSCs such as CD73, CD90 and CD105; absence of CD34 and CD45. Green open histogram represented the control, and red open histogram represented the antibodies (n=3 per group). Figure S2 Comparison of bioenergetic status between Y40‐ and Y74‐ADSCs. (A) Mitochondrial distribution and representative TEM image of intracellular mitochondria in 740‐ADSCs. Scale bar, 10 μm and 0.2 μm, respectively. (B) The total amount of ATP produced from Y40‐ADSCs and Y74‐ADSCs with a population of 1 x 10^6^ cells. (C) Relative quantification of the copy numbers of ND1/SLCO2B1 and ND5/SERPINA1 by RT‐PCR in Y40‐ADSCs and Y74‐ADSCs. ND1 and ND5 pairs for the detection of mitochondrial DNA (mtDNA), and SLCO2B1 and SERPINA1 pairs for the detection of nuclear DNA (nDNA). Significantly different (one‐way ANOVA): ns, not significant. Figure S3 Validation of mitochondrial isolation. (A) Flow cytometric analysis confirmed the complete cell disruption after chemical and mechanical lysis. (B) Quantification of mitochondrial DNA (mtDNA) isolated from different cell number. (C) Quantification of mitochondrial protein (mito‐protein) isolated from different cell number. (D) Mitochondrial ATP was kept constant at 6.2 μM in per unit of mito‐protein. Significantly different (one‐way ANOVA): ns, not significant, **P < 0.01, and ***P < 0.001. Figure S4 Evolution of oxidative stress by levels of 8‐OHdG in Y74‐ADSCs after mitochondrial uptake (n=3). Significantly different (one‐way ANOVA): ns, not significant. Figure S5 The improved cell‐migration of Y74‐ADSCs after mitochondrial uptake. (A) and (B) Representative images and quantification of control Y74‐ADSCs and mito transferred Y74‐ADSCs cell‐migration (n=3). Scale bars, 100 μm. Figure S6 The superior cell‐invasion of Y74‐ADSCs after mitochondrial uptake. (A) and (B) Representative images and quantification of cont [file BTM2-7-e10250-s006.docx]
