# Supplementary material for: In‐cytoplasm mitochondrial transplantation for mesenchymal stem cells engineering and tissue regeneration
Source: Bioeng Transl Med. 2021 Sep 28;7(1):e10250. doi: 10.1002/btm2.10250 (PMC8780934; doi:10.1002/btm2.10250)
Supplement: Supplementary file 7 — Table S1 List of 58 highly expressed proteins in mito‐transferred Y74‐ADSCs CM as compared to control Y74‐ADSCs CM (log2FC > 2). [file BTM2-7-e10250-s007.docx]

**Supplementary table legends**

Table S1 List of 58 highly expressed proteins in mito-transferred Y74-ADSCs CM as compared to control Y74-ADSCs CM (log_2_FC > 2).

Table S1

| Number | Symbol | Description | Log_2_FC | P Value |
| --- | --- | --- | --- | --- |
| 1 | FLNA | Filamin A | 74.2 | 9.67E-05 |
| 2 | PLEC | Plectin | 51.9 | 2.76E-07 |
| 3 | SPTAN1 | Spectrin Alpha, Non-Erythrocytic 1 | 46.3 | 1.99E-05 |
| 4 | FLNC | Filamin C | 41.8 | 1.67E-06 |
| 5 | FLNB | Filamin B | 33.6 | 8.20E-06 |
| 6 | TLN1 | Talin 1 | 33.1 | 0.00011 |
| 7 | TAGLN | Transgelin | 31.7 | 0.00058 |
| 8 | ENO1 | Enolase 1 | 31.5 | 0.000562 |
| 9 | ACTN1 | Actinin Alpha 1 | 29.1 | 0.00052 |
| 10 | SEMA7A | Semaphorin 7A | 26.3 | 0.000307 |
| 11 | ALCAM | Activated Leukocyte Cell Adhesion Molecule | 25.5 | 1.77E-05 |
| 12 | LMNA | Lamin A/C | 25.4 | 0.000785 |
| 13 | PPIA | Peptidylprolyl Isomerase A | 25.0 | 0.000168 |
| 14 | HNRNPA2B1 | Heterogeneous Nuclear Ribonucleoprotein A2/B1 | 24.3 | 0.000749 |
| 15 | MMP14 | Matrix Metallopeptidase 14 | 23.8 | 8.49E-06 |
| 16 | TAGLN2 | Transgelin 2 | 15.9 | 0.00164 |
| 17 | EEF2 | Eukaryotic Translation Elongation Factor 2 | 15.4 | 0.00122 |
| 18 | ANPEP | Alanyl Aminopeptidase | 15.3 | 0.000212 |
| 19 | EZR | Ezrin | 14.9 | 0.020949 |
| 20 | TXNRD1 | Thioredoxin Reductase 1 | 14.6 | 0.000416 |

Table S1 continued

| Number | Symbol | Description | Log_2_FC | P Value |
| --- | --- | --- | --- | --- |
| 21 | SERPINE1 | Serpin Family E Member 1 | 14.6 | 0.029534 |
| 22 | MYL9 | Myosin Light Chain 9 | 13.6 | 0.001011 |
| 23 | DPYSL2 | Dihydropyrimidinase Like 2 | 12.8 | 0.012498 |
| 24 | PFN1 | Profilin 1 | 12.6 | 0.000676 |
| 25 | YWHAB | Tyrosine 3-Monooxygenase/Tryptophan 5-Monooxygenase Activation Protein Beta | 11.8 | 0.010822 |
| 26 | RRBP1 | Ribosome Binding Protein 1 | 11.7 | 0.002527 |
| 27 | S100A10 | S100 Calcium Binding Protein A10 | 11.7 | 0.006709 |
| 28 | LRP1 | LDL Receptor Related Protein 1 | 11.6 | 0.024628 |
| 29 | CFL1 | Cofilin 1 | 11.1 | 0.020266 |
| 30 | MMP3 | Matrix Metallopeptidase 3 | 10.7 | 0.000481 |
| 31 | SPTBN1 | Spectrin Beta, Non-Erythrocytic 1 | 23.5 | 1.22E-05 |
| 32 | HLA-A | Major Histocompatibility Complex, Class I, A | 22.7 | 0.007414 |
| 33 | LMO7 | LIM Domain 7 | 22.6 | 0.000159 |
| 34 | PKM | Pyruvate Kinase M1/2 | 21.2 | 0.004322 |
| 35 | S100A11 | S100 Calcium Binding Protein A11 | 20.9 | 1.75E-05 |
| 36 | VCL | Vinculin | 20.2 | 0.009203 |
| 37 | MSN | Moesin | 19.7 | 0.003233 |
| 38 | YWHAZ | Tyrosine 3-Monooxygenase/Tryptophan 5-Monooxygenase Activation Protein Zeta | 19.2 | 0.000794 |
| 39 | EEF1A1 | Eukaryotic Translation Elongation Factor 1 Alpha 1 | 18.8 | 6.34E-05 |
| 40 | IQGAP1 | IQ Motif Containing GTPase Activating Protein 1 | 18.5 | 6.26E-05 |
| 41 | HNRNPA1 | Heterogeneous Nuclear Ribonucleoprotein A1 | 18.5 | 4.80E-05 |
| 42 | CTSD | Cathepsin D | 17.4 | 0.00172 |
| 43 | THBS1 | Thrombospondin 1 | 16.8 | 0.037006 |
| 44 | MYL12B | Myosin Light Chain 12B | 16.6 | 0.000233 |
| 45 | TPI1 | Triosephosphate Isomerase 1 | 9.4 | 0.04573 |

Table S1 continued

| Number | Symbol | Description | Log_2_FC | P Value |
| --- | --- | --- | --- | --- |
| 46 | PRDX1 | Peroxiredoxin 1 | 8.9 | 0.003719 |
| 47 | CSPG4 | Chondroitin Sulfate Proteoglycan 4 | 8.2 | 0.012738 |
| 48 | UBA1 | Ubiquitin Like Modifier Activating Enzyme 1 | 7.6 | 0.013177 |
| 49 | YWHAQ | Tyrosine 3-Monooxygenase/Tryptophan 5-Monooxygenase Activation Protein Theta | 7.4 | 0.007468 |
| 50 | GDI2 | GDP Dissociation Inhibitor 2 | 7.3 | 0.03734 |
| 51 | AKAP2 | A-Kinase Anchoring Protein 2 | 7.1 | 0.027945 |
| 52 | MAP4 | Microtubule Associated Protein 4 | 7.0 | 0.02197 |
| 53 | SERPINH1 | Serpin Family H Member 1 | 7.0 | 0.039072 |
| 54 | ACLY | ATP Citrate Lyase | 6.9 | 0.010404 |
| 55 | FASN | Fatty Acid Synthase | 6.7 | 0.017014 |
| 56 | STIP1 | Stress Induced Phosphoprotein 1 | 6.3 | 0.025698 |
| 57 | GLUD1 | Glutamate Dehydrogenase 1 | 5.8 | 0.020844 |
| 58 | SUMF2 | Sulfatase Modifying Factor 2 | 4.4 | 0.040244 |
